# Characterization of cerebrospinal fluid DNA methylation age during the acute recovery period following aneurysmal subarachnoid hemorrhage

**DOI:** 10.1186/s43682-021-00002-6

**Published:** 2021-12-20

**Authors:** Lacey W. Heinsberg, Dongjing Liu, John R. Shaffer, Daniel E. Weeks, Yvette P. Conley

**Affiliations:** 1 Department of Human Genetics, Graduate School of Public Health, University of Pittsburgh, Public Health 3102A, 130 De Soto Street, Pittsburgh, PA 15261, USA.; 2 Department of Genetics and Genomic Sciences, Icahn School of Medicine at Mount Sinai, New York, NY, USA.; 3 Department of Oral Biology, School of Dental Medicine, University of Pittsburgh, Pittsburgh, PA, USA.; 4 Department of Biostatistics, Graduate School of Public Health, University of Pittsburgh, Pittsburgh, PA, USA.; 5 Department of Health Promotion and Development, School of Nursing, University of Pittsburgh, Pittsburgh, PA, USA.

**Keywords:** Stroke, Epigenomics, Epigenetics, Age acceleration, Group-based trajectory analysis

## Abstract

**Background:**

Biological aging may occur at different rates than chronological aging due to genetic, social, and environmental factors. DNA methylation (DNAm) age is thought to be a reliable measure of accelerated biological aging which has been linked to an array of poor health outcomes. Given the importance of chronological age in recovery following aneurysmal subarachnoid hemorrhage (aSAH), a type of stroke, DNAm age may also be an important biomarker of outcomes, further improving predictive models. Cerebrospinal fluid (CSF) is a unique tissue representing the local central nervous system environment post-aSAH. However, the validity of CSF DNAm age is unknown, and it is unclear which epigenetic clock is ideal to compute CSF DNAm age, particularly given changes in cell type heterogeneity (CTH) during the acute recovery period. Further, the stability of DNAm age post-aSAH, specifically, has not been examined and may improve our understanding of patient recovery post-aSAH. Therefore, the purpose of this study was to characterize CSF DNAm age over 14 days post-aSAH using four epigenetic clocks.

**Results:**

Genome-wide DNAm data were available for two tissues: (1) CSF for *N* = 273 participants with serial sampling over 14 days post-aSAH (*N =* 850 samples) and (2) blood for a subset of *n* = 72 participants at one time point post-aSAH. DNAm age was calculated using the Horvath, Hannum, Levine, and “Improved Precision” (Zhang) epigenetic clocks. “Age acceleration” was computed as the residuals of DNAm age regressed on chronological age both with and without correcting for CTH. Using scatterplots, Pearson correlations, and group-based trajectory analysis, we examined the relationships between CSF DNAm age and chronological age, the concordance between DNAm ages calculated from CSF versus blood, and the stability (i.e., trajectories) of CSF DNAm age acceleration over time during recovery from aSAH. We observed moderate to strong correlations between CSF DNAm age and chronological age (*R* = 0.66 [Levine] to *R* = 0.97 [Zhang]), moderate to strong correlations between DNAm age in CSF versus blood (*R* = 0.69 [Levine] to *R* = 0.98 [Zhang]), and stable CSF age acceleration trajectories over 14 days post-aSAH in the Horvath and Zhang clocks (unadjusted for CTH), as well as the Hannum clock (adjusted for CTH).

**Conclusions:**

CSF DNAm age was generally stable post-aSAH. Although correlated, CSF DNAm age differs from blood DNAm age in the Horvath, Hannum, and Levine clocks, but not in the Zhang clock. Taken together, our results suggest that, of the clocks examined here, the Zhang clock is the most robust to CTH and is recommended for use in complex tissues such as CSF.

## Background

Across the spectrum of neurological injury populations, identifying therapeutic targets of intervention to improve patient outcomes has been a challenge. The aneurysmal subarachnoid hemorrhage (aSAH) population is no exception. After aSAH, while extreme variability in patient recovery is observed, younger patients generally do better following injury [1] underscoring the importance of chronological age as a predictor of outcomes. However, given within-individual variability such as genomic, social, and environmental factors, it is thought that “bio-logical aging” for many individuals happens at different rates and that chronological age is often a flawed surrogate measure of this phenomenon. For this reason, a substantial amount of work has been dedicated to identifying molecular biomarkers of aging. One of the most promising thus far is DNA methylation (DNAm) age which can be computed from “epigenetic clocks” and is suggested to be applicable across the lifespan and in all sources of biological tissues [2].

Several epigenetic clocks have been proposed over the last decade including the Horvath [3, 4], Hannum [5], Levine [6], and “Improved Precision” (i.e., Zhang) [7] clocks which use DNAm data from 353, 71, 513, and 514 CpG sites, respectively. DNAm age estimated by all four epigenetic clocks is strongly correlated with chronological age despite important differences in clock construction detailed below. Individuals with a DNAm age greater than their chronological age are said to have “age acceleration” which has been associated with many negative health outcomes such as cancer [8], Parkinson’s disease [9], cardiovascular disease [10], and all-cause mortality [11]. While the Horvath, Hannum, and Zhang clocks were developed to estimate chronological age, the Levine clock expanded on this to estimate a biological age metric known as “phenotypic age,” which was based not only on chronological age, but also other biological factors predictive of mortality (e.g., albumin, creatinine) [6]. Further, the Horvath clock was specifically developed to be a “pan tissue” clock by using training datasets with DNAm data generated from many biological tissues (e.g., brain, kidney, blood) whereas the Hannum and Levine clocks were developed using only DNAm data generated from the blood (though they have been subsequently examined and validated in other tissues). Of the clocks mentioned here, the Zhang clock was developed most recently and was designed to outperform all others as it was developed using training data from 13,661 blood and saliva samples, a number that far exceeds the sample sizes of its predecessors. To better understand epigenetic aging, an expanded investigation of clocks in diverse sets of tissues and diseases are needed, including longitudinal evaluations [12]. Although DNAm age has been examined in a wide range of biological tissues (e.g., blood, kidney, liver, tumor, brain [2]), it has not been examined in cerebrospinal fluid (CSF), a tissue that is critical for normal neuronal function; provides protection, nourishment, and local environmental regulation for the brain and spinal cord [13]; and can be used for clinical analyses.

Under normal physiological conditions, CSF is clear and contains ions, vitamins, and very few cells (less than five cells per milliliter) [13]. Following aSAH, however, blood accumulates in the subarachnoid space and mixes with CSF [14]. The neuronal response to this contamination is immediate degradation of hemoglobin, resulting in an increase in reactive oxygen species, cellular damage/repair, inflammation, and an acute immune response [15] which often leads to secondary injuries that could impact DNAm age [16, 17]. Because DNAm is dynamic and responsive to external stimuli [18], and that CSF composition and secretion are finely regulated and renewed approximately four times every day [13], peripheral cell types may behave differently in this new environment, potentially resulting in cellular reprogramming, polycreodism, and DNAm patterns not typically observed in the blood [19]. Further, while the peripheral blood contaminates the CSF following aSAH, it gradually clears during recovery. Likewise, cell types originating in the brain (e.g., ependymal) and ruptured vessel can be observed in post-aSAH CSF [13, 20]. As such, in many cases of neurologic injury where CSF is drained as part of clinical management to reduce intracranial pressure, including aSAH, this tissue may support an improved understanding of the local environment of the central nervous system. Trajectories of age acceleration during recovery from neurologic injury may offer insight into the stability of DNAm age in acute pathological conditions such as aSAH and improve our understanding of both DNAm age and recovery post-aSAH. Despite this, the validity and potential utility of DNAm age computed using CSF is not understood, which is an important gap in our knowledge.

Therefore, the purpose of this longitudinal, observational study was to characterize CSF DNAm age over the immediate 14 day recovery period following aSAH. As part of this characterization, we wanted to better understand the relationships between CSF DNAm age and chronological age, the concordance between DNAm ages calculated using CSF versus peripheral blood, the stability (i.e., trajectories) of CSF age acceleration during recovery from aSAH, and the correlations between four epigenetic clocks (Horvath [3, 4], Hannum [5], Levine [6], and Zhang [7]). Given our focus on CSF in a pathological condition, a critical piece of this study included examination of the effects of cell-type heterogeneity (CTH) as cell-type proportions can vary across time, tissues, and individuals and can impact DNAm [21]. Therefore, all of our analyses were conducted both with and without considering the effects of CTH to better understand how CTH impacts DNAm age as a whole in CSF, a complex tissue.

## Results

### Sample characteristics

Our final sample size consisted of *n* = 273 aSAH participants (*n* = 850 observations). All participants had CSF DNAm data at up to five cross-sectional time points over 14 days post-aSAH including time 1 (days 0 to 2), time 2 (days 3 to 5), time 3 (days 6 to 8), time 4 (days 9 to 11), and time 5 (days 12 to 14). Of the overall sample, *n* = 72 participants also had blood DNAm data available at cross-sectional time point 1 (days 0 to 2). Sample characteristics are presented (Table 1). Our overall sample (*n* = 273) had a mean (± standard deviation) age of 52.9 (± 11.1) years and was 68.5% female and 87.2% White with Fisher grades of 2, 3, or 4 accounting for 29.7%, 49.5%, and 20.9% of the sample, respectively. The mean body mass index (BMI) was 28.1 (± 7.2) kg/m^2^, and 53.8% of participants were active smokers. We observed similar statistics in the subset of participants with both CSF and blood DNAm data available on days 0 to 2 post-aSAH (*n* = 72). The sample characteristics observed were comparable to statistics observed in the general aSAH population [22].

### Correlation between DNAm age and chronological age

**Fig. 1:**
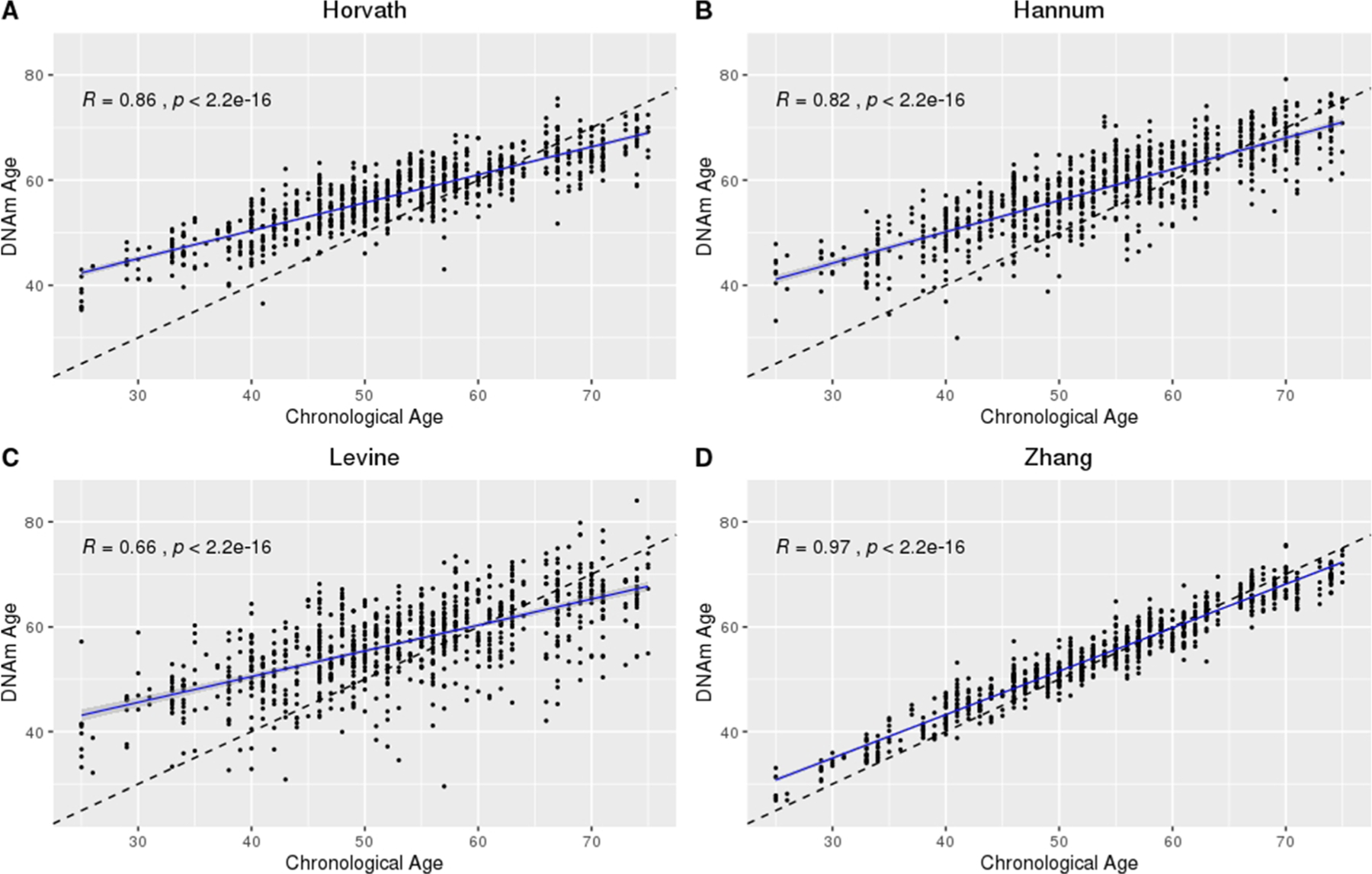
Chronological age versus DNAm age in CSF on days 0 to 14 post-aSAH using the Horvath, Hannum, Levine, and Zhang epigenetic clocks. **A** Horvath. **B** Hannum. **C** Levine. **D** Zhang. Sample size, *n* = 273 at up to 5 time points (*N* = 850 observations over 14 days post-aSAH); dashed line, *x* = *y*; solid line, predicted model fit. DNAm, DNA methylation; CSF, cerebrospinal fluid; aSAH, aneurysmal subarachnoid hemorrhage; *R*, correlation computed using Pearson method

Across all CSF samples (*n* = 273 at up to five time points over 14 days post-aSAH), DNAm age was moderately to strongly correlated with chronological age in the Horvath (*R* = 0.86, *p* < 2.2E−16), Hannum (*R* = 0.82, *p* < 2.2E−16), Levine (*R* = 0.66, *p* < 2.2E−16), and Zhang (*R* = 0.97, *p* < 2.2E−16) clocks (Fig. 1). The relationship between DNAm age and chronological age was similar for the Horvath, Hannum, and Levine clocks and strongest in the Zhang clock (Fig. S1). We noted between participant variation and that the relationship between CSF DNAm age and chronological age differed as a function of chronological age, most notably in the Horvath, Hannum, and Levine clocks (Fig. 1). Specifically, we observed higher DNAm age than expected in younger participants and lower DNAm age than expected in older participants. Within cross-sectional time points, we observed similar correlations between chronological age and DNAm age in CSF over time, with the strongest correlations observed in the Zhang clock (Figs. S2, S3, S4, and S5).

**Fig. 2:**
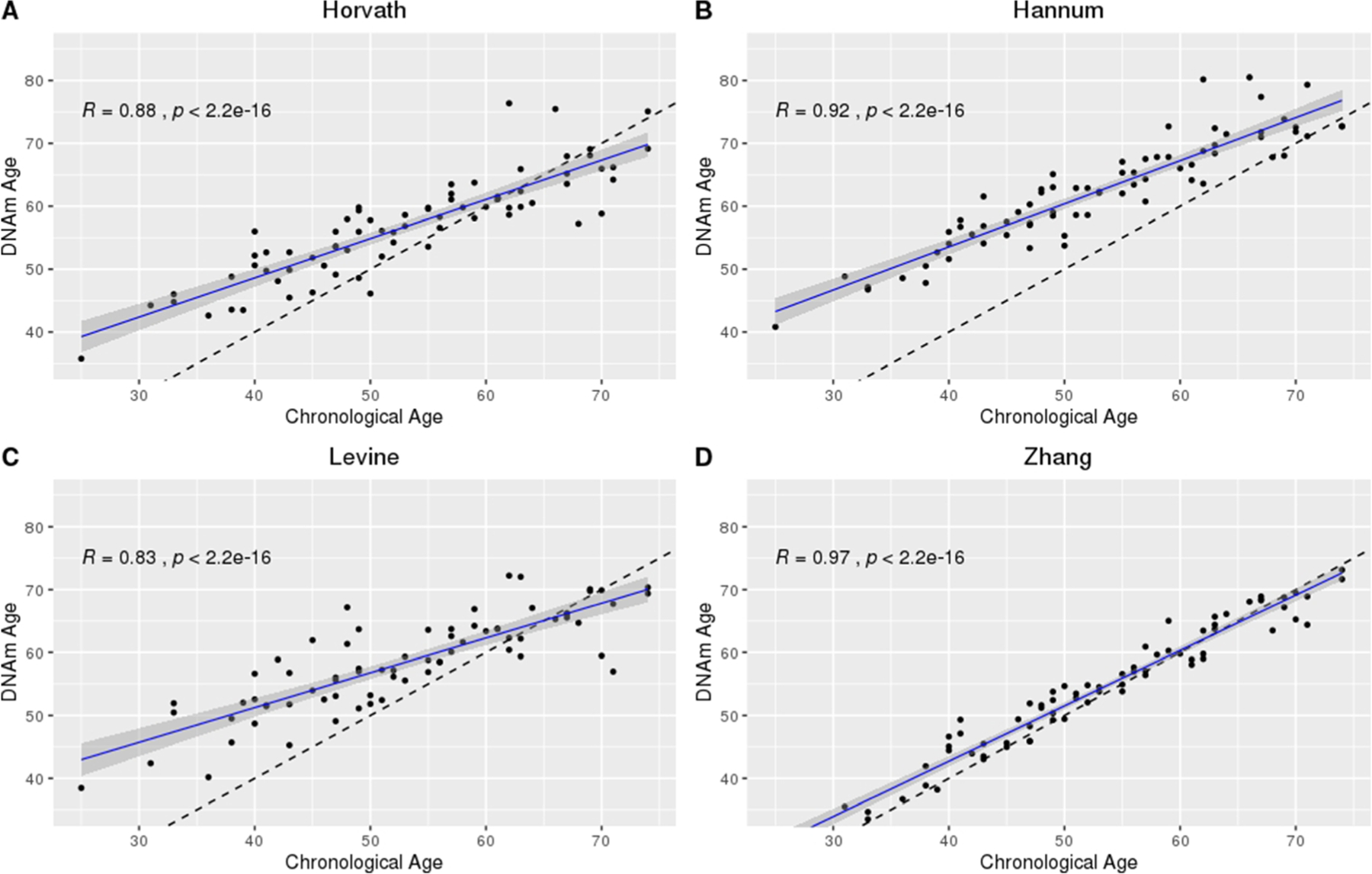
Chronological age versus DNAm age in the blood at time 1 (days 0 to 2) post-aSAH using the Horvath, Hannum, Levine, and Zhang epigenetic clocks. **A** Horvath. **B** Hannum. **C** Levine. **D** Zhang. Sample size, *n* = 72 with both CSF and blood DNA methylation data at cross-sectional time point 1 (days 0 to 2 post-aSAH); dashed line, *x* = *y*; solid line, predicted model fit. DNAm, DNA methylation; aSAH, aneurysmal subarachnoid hemorrhage; *R*, correlation computed using Pearson method

Within the subset of participants for which blood was available (*n* = 72 on days 0 to 2 post-aSAH), chronological age was strongly correlated with DNAm age in the Horvath (*R* = 0.88, *p* < 2.2E−16), Hannum (*R* = 0.92, *p* < 2.2E−16), Levine (*R* = 0.83, *p* < 2.2E−16), and Zhang (*R* = 0.97, *p* < 2.2E−16) clocks (Fig. 2). The relationship between DNAm age and chronological age was similar for the Horvath, Hannum, and Levine clocks and strongest in the Zhang clock (Fig. S6). Correlations between chronological age and DNAm age were stronger in blood compared with CSF for the Horvath, Hannum, and Levine clocks but were the same for the Zhang clock. In all clocks, the relationship between DNAm age and chronological age again differed as a function of chronological age.

### Correlation between DNAm age and age acceleration in CSF and blood

**Fig. 3:**
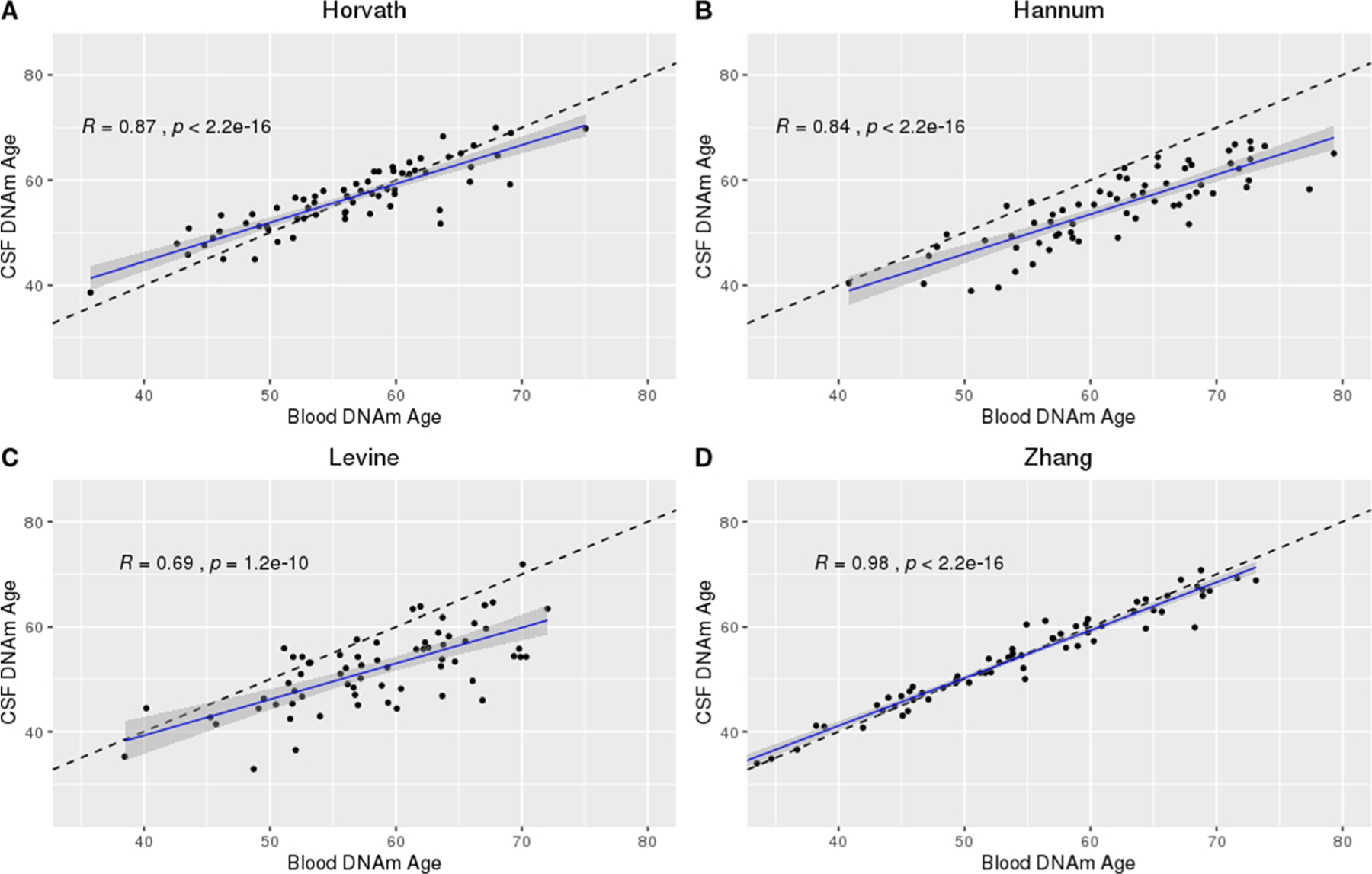
DNAm age in CSF versus blood at time 1 (days 0 to 2) post-aSAH using the Horvath, Hannum, Levine, and Zhang epigenetic clocks. **A** Horvath. **B** Hannum. **C** Levine. **D** Zhang. Sample size, *n* = 72 with both CSF and blood DNA methylation data at cross-sectional time point 1 (days 0 to 2 post-aSAH); dashed line, *x* = *y*; solid line, predicted model fit. DNAm, DNA methylation; CSF, cerebrospinal fluid; aSAH, aneurysmal subarachnoid hemorrhage; *R*, correlation computed using Pearson method

Within the subset of participants for which both CSF and blood DNAm data were available (*n* = 72 on days 0 to 2 post-aSAH), we observed moderate to strong correlations between DNAm ages measured in CSF versus blood in the Horvath (*R* = 0.87, *p* < 2.2E−16), Hannum (*R* = 0.84, *p* < 2.2E−16), Levine (*R* = 0.69, *p* = 1.2E −10), and Zhang (*R* = 0.98, *p* < 2.2E−16) clocks (Fig. 3).

**Fig. 4:**
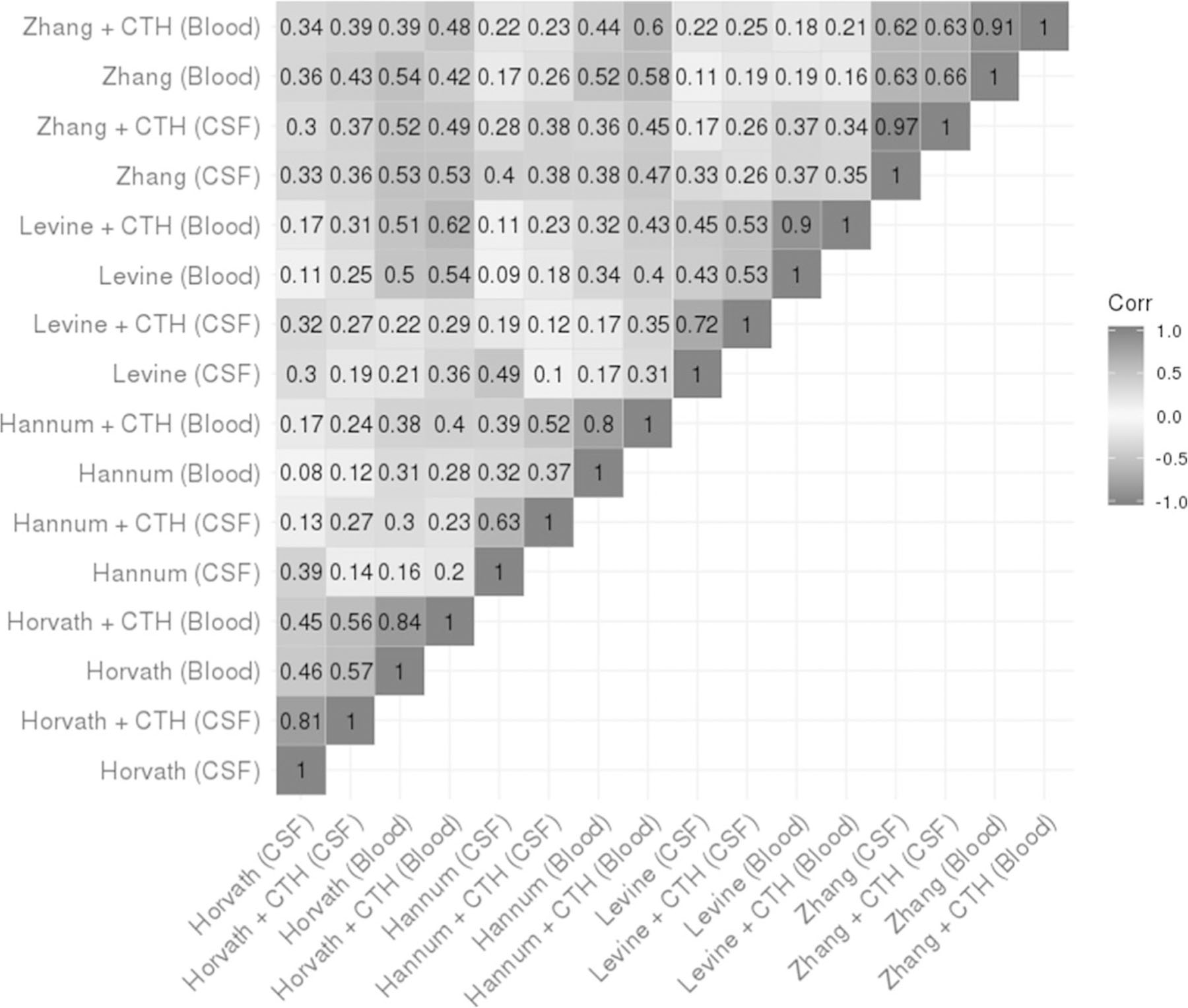
Correlation heatmap of unadjusted and CTH-adjusted age acceleration at time 1 (days 0 to 2) post-aSAH computed in CSF and blood using the Horvath, Hannum, Levine, and Zhang epigenetic clocks. Sample size, *n* = 72 with both CSF and blood DNA methylation data at cross-sectional time point 1 (days 0 to 2 post-aSAH). CSF, cerebrospinal fluid; aSAH, aneurysmal subarachnoid hemorrhage; CTH, cell-type heterogeneity. All values presented are *R* values indicating age acceleration correlation computed using Pearson method

Next, we used the subset of participants with both CSF and blood available (*n* = 72 at cross-sectional time point 1 on days 0 to 2 post-aSAH) to compare DNAm age (Fig. S7) and age acceleration (Fig. 4) computed from all clocks with optional correction for CTH. Correlations between DNAm age in all clocks and tissues ranged from *R* = 0.60 (Levine [CSF] and Zhang [blood]) to *R* = 0.98 (Zhang [CSF] and Zhang [blood]) (Fig. S7). Correlations between age acceleration in all clocks ranged from *R* = 0.08 (Hannum [blood] and Horvath [CSF]) to as large as *R* = 0.97 (Zhang [CSF] and Zhang [CSF + CTH]) (Fig. 4). CSF CTH data used to compute the age acceleration metric adjusted for CTH are presented graphically (Figs. S8 and S9).

**Fig. 5:**
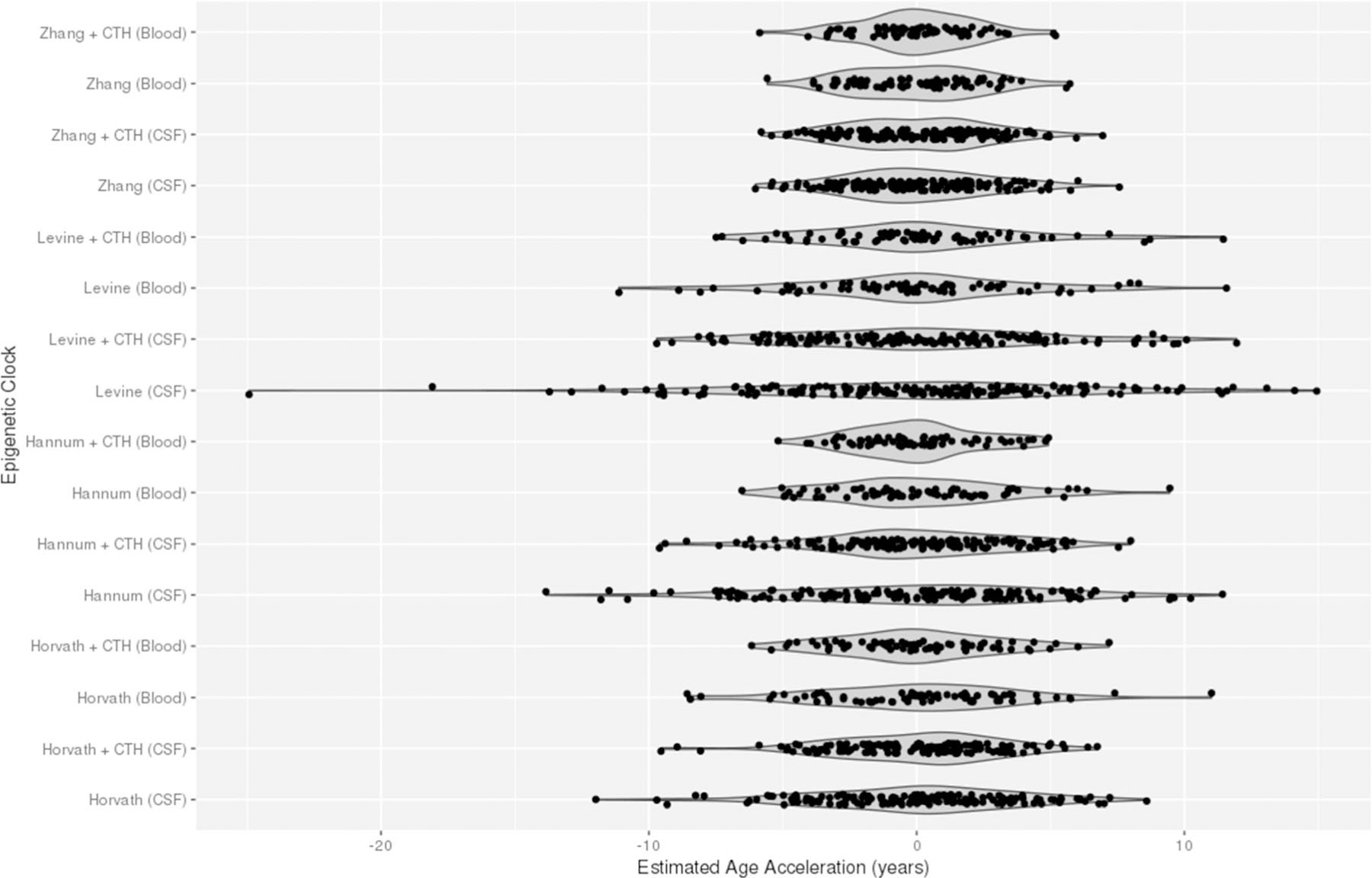
Sina plots of unadjusted and CTH-adjusted age acceleration at time 1 (days 0 to 2) post-aSAH computed in CSF and blood using the Horvath, Hannum, Levine, and Zhang epigenetic clocks. Sample size, *n* = 72 with both CSF and blood DNA methylation data at cross-sectional time point 1 (days 0 to 2 post-aSAH). CSF, cerebrospinal fluid; aSAH, aneurysmal subarachnoid hemorrhage; CTH, cell-type heterogeneity

Finally, we compared the age acceleration data distributions and densities between clocks with optional correction for CTH (Fig. 5). Levine CSF age acceleration had the widest range of values while Hannum CTH-adjusted blood age acceleration had the narrowest range of values. Of the four clocks, the Zhang clock data distributions looked most similar regardless of tissue and CTH-adjustment.

### Trajectories of CSF age acceleration

#### Horvath clock

**Fig. 6:**
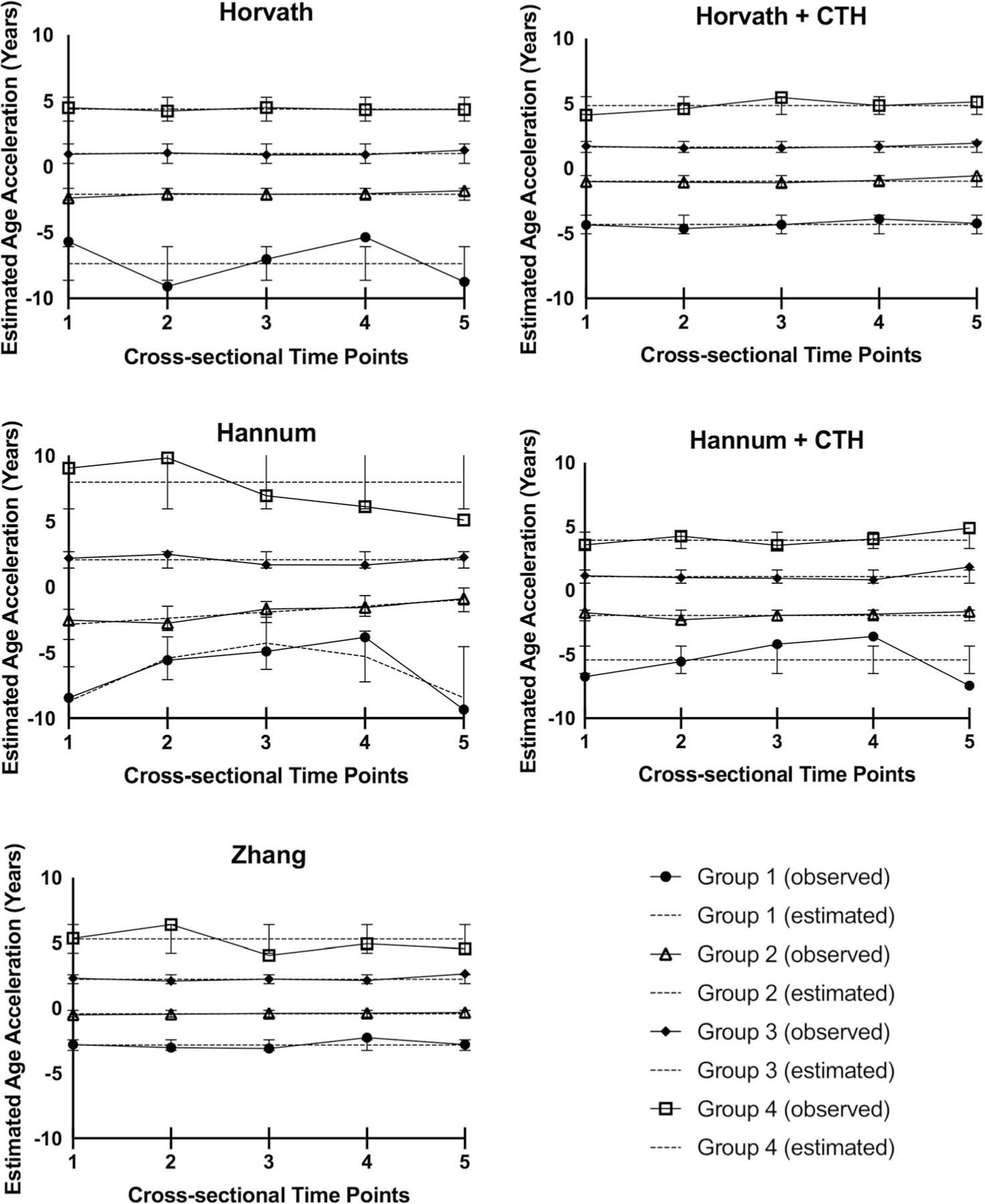
Age acceleration trajectory plots for Horvath, Hannum, and Zhang epigenetic clocks. Note that the above plots portray inferred trajectory groups and are not directly comparable as the group membership changes between plots as shown in Table 2; *n* = 273 at up to five time points (*N* = 850 observations over 14 days post-aSAH condensed to 5 cross-sectional time points); times listed correspond to cross-sectional time points (time 1 [days 0 to 2], time 2 [days 3 to 5], time 3 [days 6 to 8], time 4 [days 9 to 11], time 5 [days 12 to 14]); 95% confidence intervals shown are for estimated trajectories and not observed trajectories. CSF, cerebrospinal fluid; CTH, cell-type heterogeneity; aSAH, aneurysmal subarachnoid hemorrhage. Note that the Levine trajectory models and Zhang CTH-adjusted model did not pass posterior quality control and are presented in the Supplementary Material

Finally, in an effort to understand the stability (i.e., trajectories) of CSF age acceleration over time during recovery from aSAH, we used group-based trajectory analysis (GBTA) to examine age acceleration over time (both with and without adjusting for CTH). Inferred age acceleration trajectory groups for the Horvath (adjusted and unadjusted for CTH), Hannum (adjusted and unadjusted for CTH), and Zhang (unadjusted for CTH) clocks are presented (Fig. 6). As discussed in more detail below, the trajectory models for the Levine clock (both unadjusted and adjusted for CTH) and the Zhang clock (adjusted for CTH) did not pass posterior model quality control (QC), so are not included in Fig. 6. For age acceleration data computed using the Horvath clock, both unadjusted and adjusted for CTH, four distinct, flat trajectory groups (groups 1 through 4) were inferred, suggesting that Horvath DNAm age acceleration did not change over time during recovery from aSAH (Fig. 6, Horvath and Horvath + CTH). All unadjusted and CTH-adjusted model selection parameters including the Bayesian Information Criterion (BIC) computed from iterative model testing as well as posterior model QC indices are presented (Tables S2a, S2b, S3a, and S3b).

#### Hannum clock

For age acceleration data unadjusted for CTH computed using the Hannum clock, we again inferred four distinct trajectory groups. While the two groups with the highest age acceleration (groups 3 and 4) did not change over time, we observed a slow increase in age acceleration in group 2 and an increase followed by a return to baseline in group 1 (Fig. 6, Hannum). When we controlled for CTH in the calculation of age acceleration, this temporal variation was washed out resulting in four flat trajectory groups with no change over time (Fig. 6, Hannum + CTH). All model selection parameters including the BIC computed from iterative model testing as well as posterior model QC indices are presented (Tables S4a, S4b, S5a, and S5b). It should be noted that the plots in Fig. 6 depict inferred trajectory groups and are not directly comparable because group membership changes after adjustment for CTH as shown in Table 2 (e.g., in Fig. 6, Hannum, group 1 has only 8 participants while in Fig. 6, Hannum + CTH, Group 1 has 23 participants).

#### Levine clock

GBTA plots for the Levine clock are presented (Fig. S10). Neither the trajectory model unadjusted for CTH nor the trajectory model adjusted for CTH passed QC procedures due to inadequate odds of correct classification of the middle groups. In other words, while we were confident in group participant assignment in the highest and lowest DNAm groups (groups 4 and 1, respectively), participant assignment could not be distinguished with high confidence for the middle groups. All model selection parameters including BIC computed from iterative model testing as well as posterior model QC indices are presented (Tables S6a, S6b, S7a, and S7b).

#### Zhang clock

For age acceleration data unadjusted for CTH computed using the Zhang clock, we inferred four distinct trajectory groups with no change over time (Fig. 6, Zhang). When we controlled for CTH in the calculation of age acceleration, the trajectory model for the Zhang clock did not pass our posterior model QC, again due to a low odds of correct classification (Fig. S10). All model selection parameters including the BIC computed from iterative model testing as well as posterior model QC indices are presented (Tables S8a, S8b, S9a, and S9b).

#### Characterization of trajectory groups (Horvath, Hannum, and Zhang)

Next, we computed participant characteristics for identified trajectory groups for the trajectory models that passed posterior QC (Horvath, Hannum, and Zhang [unadjusted for CTH]). For all clocks, we noticed a difference between sexes with a decreasing proportion of females as age acceleration increased, though this was only statistically significant in the Hannum clock (*p* < 0.0001). This is particularly notable in the age acceleration trajectory groups unadjusted for CTH computed using the Hannum clock. The group with the lowest age acceleration (group 1) was 93.3% female while the group with the highest age acceleration (group 4) was only 16.7% female. We observed no other differences in participant characteristics by trajectory group.

### Bivariate associations between DNAm age acceleration and participant characteristics

Lastly, we wanted to understand if DNAm age acceleration was associated with participant characteristics independent of inferred trajectory groups (Table 3). We observed associations between sex and Horvath CSF DNAm age acceleration (*p* = 0.02), Hannum CSF DNAm age acceleration (*p* < 0.0001), and Hannum CSF DNAm age acceleration controlling for CTH (*p* = 0.0001). We also observed associations between race and Hannum DNAm age acceleration in the blood (*p* = 0.04), Levine CSF DNAm age acceleration (*p* = 0.03), and Levine CSF DNAm age acceleration controlling for CTH (*p* = 0.003). Finally, we observed an association between smoking and Levine CSF DNAm age acceleration controlling for CTH (*p* = 0.003).

## Discussion

This study is the first to characterize CSF DNAm age over the first 14 days post-aSAH. While we observed similarities between the tissues and epigenetic clocks applied, the Zhang clock outperformed the Horvath, Hannum, and Levine clocks in a complex tissue and pathological state, living up to its name as the “Improved Precision” clock. Specifically, of the four clocks examined, the Zhang clock was the most robust to systematic differences in DNAm age by chronological age discussed in detail elsewhere [23] (Fig. 1, CSF; Fig. 2, blood). Furthermore, while we observed generally strong correlations between DNAm ages measured in CSF versus blood (Fig. 3), we observed a near perfect correlation in the Zhang clock (*R* = 0.98). Likewise, neither tissue nor CTH made a substantial difference in the distribution of the data from the Zhang clock, further supporting the clock’s robustness. Although the relationship between chronological age and DNAm age was generally steady in CSF over the five cross-sectional time points examined, we observed trending time-dependent changes in the Horvath, Hannum, and Levine clocks but not in the Zhang clock (Figs. S2 through S5). While it is somewhat surprising that the clock performed so well despite being developed in non-CSF tissues, the performance of the clock can likely be credited to its development in the largest training data set to date [7].

CSF DNAm training data was not used in the development of any of the clocks we examined. While this appears to be a potential source of variability in the Horvath, Hannum, and Levine clocks, it did not impact our results when using the Zhang clock. This is a particularly notable finding and relevant for researchers using DNAm data from complex tissues such as CSF. Specifically, post-aSAH in particular, CTH requires careful consideration as CSF is heavily contaminated with blood immediately following aneurysm rupture but gradually clears over time during recovery. As discussed below, no reference-based method for cell type deconvolution exists for CSF DNAm data. While we carefully controlled for CTH using a reference-free method [24], it would be interesting to compare our CTH-adjusted results to a reference-based method developed specifically for CSF post-aSAH. Likewise, if we had RNA sequencing data for our samples in parallel, a much more nuanced exploration of the cell types would be possible [19, 25]. While the true identities of cell types present in the CSF post-aSAH would be scientifically and clinically useful for the aSAH research community, because this study focused on characterizing DNAm age over time, direct biological interpretation of cell-type specific results was not a focus of our study. Likewise, the Zhang clock was robust to CTH, making these data unnecessary in this context.

Aside from CTH, we did not control for the influence of participant characteristics (e.g., sex, race, smoking, or BMI) in our calculation of age acceleration as justified below. We observed that sex was associated with inferred trajectory group assignment in the Hannum clock (Table 2) which is consistent with existing literature suggesting that men have higher DNAm age than women [26]. This finding was confirmed by examining the associations between participant characteristics and ungrouped age acceleration metrics independent of trajectory group (Table 3). These associations were not observed in the other clocks, however, further highlighting clock differences. A surprising observation in this study was that the trajectory groups did not have other notable differences in participant characteristics.

Although this study has many strengths, there are some limitations that should be acknowledged. First, several measurements of age acceleration are reported in the literature. Most commonly, we observed (1) Δage, defined as the difference between DNAm age and chronological age, and (2) age acceleration, defined as the residuals of DNAm age regressed on age (often with the addition of covariates such as CTH). Initially, we performed our analyses using Δage and then realized that there was a systematic difference in delta age based on chronological age as described above. In contrast, the residual-based method of computing age acceleration applied here results in a metric that has no correlation with chronological age. The downside to this method, however, is that it results in a metric that is an attribute of the group and not specific to the individual. Therefore, the residual method has a higher potential sensitivity to outlying DNAm age values, though outliers were not found to be influential in our results. Clinically, Δage may be of more interest than the residual definition of age acceleration because it could be calculated for only one participant. On this note, we also want to highlight a shift in the epigenetic age literature in which a call for disease- and tissue-specific clocks [12] is being answered (e.g., placental aging clock [27], hippocampal and cortical tissue clocks [28]). A clock specifically trained using CSF DNAm data from the acute period post-aSAH would have the greatest potential clinical utility, particularly when examining patient recovery.

An additional potential limitation of this study was that all blood samples were included on a separate plate from CSF samples, so we were unable to adjust for possible CSF-blood plate batch effects. A strength of this design, however, is that there were no chip batch effects in the blood DNAm data. Additionally, the correlation coefficient does not vary by change in origin and scale [29]. Therefore, any potential CSF-blood plate effects that differ in this manner will not distort the results of our correlational analyses, which is supported by the results comparing blood and CSF DNAm age computed using the Zhang clock (Fig. 3D). Furthermore, only a subset of 72 of our participants had blood DNAm data available making our comparisons involving blood DNAm age or age acceleration quite small. Likewise, for participants with blood available, the DNAm data were only collected at one cross-sectional time point (on days 0 to 2 post-aSAH) which prevented us from comparing the trajectories of blood age acceleration over time during recovery from aSAH with CSF. Finally, aside from the cohort studied in the present analyses, no other aSAH sample with serial CSF DNAm data exists. Therefore, we were unable to replicate our findings in an independent sample.

## Conclusion

The Zhang clock outperformed the Horvath, Hanum, and Levine clocks in post-aSAH CSF and was robust to changes in CTH. Despite being developed in non-CSF tissues, DNAm age computed from all clocks was generally accurate in post-aSAH CSF. CSF age acceleration measured in all clocks was largely stable over time during recovery from aSAH, particularly once adjusting for CTH, suggesting that DNAm age is not impacted in the acute aSAH recovery period. As such, we conclude that (1) future studies could increase power by using a single measurement from more participants, rather than generating DNAm data for each participant longitudinally, and (2) it is unlikely that CSF DNAm age acceleration from the clocks examined here offers additional predictive value for recovery post-aSAH.

## Materials and methods

### Study design, setting, and sample

This study was an observational, longitudinal, secondary data analysis that capitalized on existing genome-wide DNAm data collected from a cohort of aSAH research participants. All research protocols were approved by the Institutional Review Board of the University of Pittsburgh, and informed consent was obtained from participants as part of the larger study. Participants were prospectively recruited from UPMC Presbyterian Neuro-vascular Intensive Care Unit in Pittsburgh, Pennsylvania, between 2000 and 2013 as previously described [30]. In brief, participants were included if they were diagnosed with subarachnoid hemorrhage caused by an aneurysm rupture, were at least 18 years of age, had no history of debilitating neurological disorder, and required an external ventricular drain to reduce intracranial pressure and manage CSF as part of standard care in the hospital. As part of the larger study, (1) participants were followed over 14 days post-aSAH in the hospital as complications that are predictive of long-term outcomes can occur during this acute window (e.g., cerebral vasospasm, delayed cerebral ischemia) and (2) genome-wide DNAm data were generated as described below.

### Participant characteristic data

Participant data were extracted from the medical record and included standard demographic data (e.g., age, sex, and self-reported race), BMI and smoking history (given associations between these factors and DNAm levels [31, 32]), and Fisher grade, which is a clinical variable measuring the initial extent of aSAH injury based on the amount and distribution of blood observed on a computed tomography (CT) scan. Clinically, Fisher grades can range from 1 (no blood detected) to 4 (intraventricular or intra parenchymal blood present) [33]. Of note, all participants in this study had severe enough injury (Fisher grade > 2) to require drainage of CSF as part of their standard clinical management.

### DNA methylation data collection

DNA was extracted from two biological tissue sources including (1) CSF (for all study participants [*N* = 279] with serial sampling over 14 days after aSAH) and (2) blood (for a subset of study participants [*n* = 88] at one time point after aSAH). CSF samples from ventricular drains placed as standard of care were selected for targeted post-injury days of 1, 4, 7, 10, and 13 (± 1 day) as described elsewhere [30]. DNA was extracted from CSF using the Qiamp Midi kit (Qiagen, Valencia, CA, USA) and from blood using a simple salting out procedure [34]. All DNA was stored in 1× TE buffer at 4 °C until DNAm data collection. All samples were collected, stored, processed, and extracted using identical standardized/validated protocols. Genome-wide DNAm data were generated using the Infinium Human Methylation450 BeadChip and scanned using the Illumina iSCAN (Illumina, Incorporated, San Diego, CA, USA) at the Center for Inherited Disease Research using laboratory QC procedures described in detail [30]. Standard DNA concentration and quality checks were performed prior to data collection and all DNA carried forward for data collection was considered to be high quality and high yield. Raw genome-wide DNAm data were analyzed using Genome Studio Software (Illumina, Incorporated, San Diego, CA, USA). Our data cleaning and QC process included removal of poorly performing samples, probes, and outliers [30] as well as functional normalization and robust batch correction (i.e., chip, row, and column effects) using the funtooNorm package [35]. Of note, funtooNorm was designed to handle data gathered across time and allows for interactions between tissue types [35], making it ideal for complex tissues and serial measurements. Our final post-QC sample size consisted of *N* = 273 participants with serial CSF DNAm data over 14 days post-aSAH (*N* = 850 samples) and blood DNAm data for a subset of *n* = 72 of those participants as described below.

### Cell-type heterogeneity

Because cell-type proportions can vary across time, tissues, and individuals, and that overall DNAm levels are computed using the proportion-weighted average of the cell-type specific methylation levels, CTH should be considered carefully as a potential confounder in studies of DNAm [21]. CTH is particularly important in the current analyses because, as we discussed above, CSF post-aSAH is heavily contaminated by blood cells that could take on different properties in this new space, cells originating in the brain, and cells from the ruptured vessel, which will gradually clear causing CTH to change over time.

Careful consideration was given to our choice of cell type deconvolution. Reference-based methods to infer CTH data do not exist for CSF or for blood that is now found surrounding the brain and spinal cord. We did not feel that the application of a peripheral blood reference-based method was appropriate given the poor performance of these tools in cord blood, a tissue with similar complexities to CSF (i.e., cord blood contains all components of peripheral whole blood as well as other cell types) [36]. Thus, CTH data were generated from the genome-wide DNAm data using Houseman’s reference-free method which provided estimated proportions of five cell types for each sample [24]. While this method has been shown to result in accurate proportions of major putative cell types, similar to standard principal components analysis, the true cell type identities are not known. Cell types were plotted over time using sina with violin [37] and spaghetti plots (Figs. S8 and Figs. S9).

### DNA methylation age

DNAm age was calculated using four epigenetic clocks (Horvath [3, 4], Hannum [5], Levine [6], and Zhang [7]). These methods use linear functions and clock-specific probes and coefficients to compute DNAm age as shown in Eq. 1:

(1) $$\text{DNAmAge}=m_{0}+m_{1}\beta_{1}+m_{2}\beta_{2}+\ldots +m_{n}\beta_{n}$$

where DNAmAge is the predicted DNAm age for a given individual; *m* is a clock-specific coefficient corresponding to a clock-specific probe; β is the DNAm measurement, a beta value as measured on a 0 to 1 scale, for a clock-specific probe within a given individual; and *m*_0_ is a clock-specific model intercept. It should be noted that the Horvath method also uses an age transformation function as described [3, 4] and shown in the Supplementary Material (Additional File 1, Section 1.1). Calculations for the Horvath, Hannum, and Levine clocks were performed using a modified function from the wateRmelon package [38] in R [39] (wateRmelon: agep). The wateRmelon package supplies both Horvath and Hannum coefficients for use with the “agep” function, and we modified this function to also compute Levine DNAm age as described in detail in the Supplementary Material. Calculations for the Zhang clock were made using publicly available code [40].

DNAm age was computed using both CSF DNAm data and blood DNAm data. To allow for comparability between tissues, only clock-specific probes available in both CSF and blood were used in our analysis. Following implementation of the QC pipeline described above, for the Horvath, Hannum, Levine, and Zhang epigenetic clocks, we were missing DNAm data for 1, 3, 5, and 11 probes, respectively, as detailed in Table S1. Following calculation of DNAm age, CSF data were reshaped into five cross-sectional time points including time 1 (days 0 to 2 post-aSAH), time 2 (days 3 to 5 post-aSAH), time 3 (days 6 to 8 post-aSAH), time 4 (days 9 to 11 post-aSAH), and time 5 (days 12 to 14 post-aSAH). The vast majority of the blood samples available were collected at time 1 (days 0 to 2 post-aSAH), so blood samples collected outside of this cross-sectional time point (*n* = 16) were excluded from further analyses.

### DNA methylation age acceleration

For each of the three epigenetic clocks, we computed age acceleration defined as the residuals of DNAm age regressed on chronological age within each cross-sectional time point. We computed age acceleration both with and without adjustment for CTH, including putative cell type proportions as a covariate in our regression. Because the CTH data resulted in a proportioned phenotype which added up to one, we excluded the cell type with the lowest amount of variation within our study sample to minimize confounding the results. Age acceleration was computed both with and without adjusting for extreme outliers (DNAm age > 3 times the interquartile range), and the results were found to be concordant. Therefore, we present only the age acceleration metrics unadjusted for outliers. Additional participant factors were not included in our calculation of age acceleration but were carefully examined as described below.

### Statistical analysis

Statistical analyses were conducted using R (version 3.6.0) [39] and SAS (version 9.4, SAS Institute Incorporated, Cary, NC, USA). Demographic and clinical characteristics of our sample were examined using standard descriptive statistics. CSF and blood DNAm age computed from all three clocks was compared with chronological age using scatterplots and Pearson correlations. For participants with both CSF and blood samples, we compared the correlation between DNAm age and age acceleration both with and without adjusting for CTH using Pearson correlations and heatmaps.

Next, we examined age acceleration over time during recovery from aSAH using GBTA implemented with the Proc TRAJ macro in SAS [41, 42]. While there are several methods to perform trajectory analyses such as hierarchical modeling or latent curve analysis, these methods estimate the sample average trajectory and use covariates to explain the variability around this average. In contrast, GBTA assumes the sample is composed of distinct groups, each with a different underlying age acceleration trajectory [41, 42]. This method allows us to infer trajectory groups based solely on age acceleration while also estimating how participant characteristics differ between group membership.

GBTA was performed through iterative modeling, comparing models with varying group numbers and shapes (i.e., intercept-only, linear, and quadratic terms) to infer distinct trajectory groups. BIC was used as our primary indicator of model fit, with a larger BIC indicating a better model fit [41, 42]. Following selection of a best-fitting model, we performed a posterior QC check of the model using several model-fit indices including ensuring (1) the average posterior probability of group assignment was at least 0.7, (2) the odds of correct classification was greater than 5, and (3) the estimated group assignment percentages were approximately equal to the observed group assignment percentages [41, 42].

As described above, chronological age was adjusted for in the calculation of age acceleration. Although sex, BMI, smoking status have been shown to be associated with DNAm, we did not adjust for additional covariates during GBTA because we wanted to use a data-driven approach to characterize and identify trajectory groups based solely on age acceleration. However, following the identification of the trajectory groups, we used one-way analysis of variance and chi-square/Fisher’s exact tests to understand how participant characteristics (e.g., sex, BMI, smoking status, Fisher grade) differed between inferred trajectory groups. Finally, we used linear regression to understand the associations between participant characteristics and age acceleration metrics independent of trajectory groups.

## Supplementary Material

Supplement1 **Additional file 1: Supplementary Methods.** DNAm age calculation; **Table S1.** Summary of missing probes in CSF and blood for Horvath, Hannum, Levine, and Zhang epigenetic clocks; **Figure S1.** Regression line overlays comparing chronological age versus DNAm age in CSF over 14 days post-aSAH using the Horvath, Hannum, Levine, and Zhang epigenetic clocks; **Figure S2.** Chronological age versus DNA methylation age in CSF at cross-sectional time points over 14 days post-aSAH using the Horvath epigenetic clock; **Figure S3.** Chronological age versus DNAm age in CSF at cross-sectional time points over 14 days post-aSAH using the Hannum epigenetic clock; **Figure S4.** Chronological age versus DNAm age in CSF at cross-sectional time points over 14 days post-aSAH using the Levine epigenetic clock; **Figure S5.** Chronological age versus DNAm age in CSF at cross-sectional time points over 14 days post-aSAH using the Zhang epigenetic clock; **Figure S6.** Regression line overlays comparing chronological age versus DNAm age in blood at Time 1 (Days 0 to 2) post-aSAH using the Horvath, Hannum, Levine, and Zhang epigenetic clocks; **Figure S7.** Correlation heatmap of DNA methylation age at Time 1 (days 0 to 2) post-aSAH computed in CSF and blood using the Horvath, Hannum, Levine, and Zhang epigenetic clocks; **Figure S8.** Sina with violin plots of CSF putative cell types included in our CTH-adjusted analyses; **Figure S9.** Spaghetti plots of CSF putative cell types included in our CTH-adjusted analyses; **Figure S10.** Trajectory plots of unadjusted and CTH-adjusted CSF age acceleration from Levine and Zhang epigenetic clocks; **Table S2a.** Model selection for Horvath age acceleration group-based trajectory analysis; **Table S2b.** Horvath age acceleration trajectory group posterior model quality control evaluation; **Table S3a.** Model selection for CTH-adjusted Horvath age acceleration group-based trajectory analysis; **Table S3b.** Horvath CTH-adjusted age acceleration trajectory group posterior model quality control evaluation; **Table S4a.** Model selection for Hannum age acceleration group-based trajectory analysis; **Table S4b.** Hannum age acceleration trajectory group posterior model quality control evaluation; **Table S5a.** Model selection for CTH-adjusted Hannum age acceleration group-based trajectory analysis; **Table S5b.** Hannum CTH-adjusted age acceleration trajectory group posterior model quality control evaluation; **Table S6a.** Model selection for Levine age acceleration group-based trajectory analysis; **Table S6b.** Levine age acceleration trajectory group posterior model quality control evaluation; **Table S7a.** Model selection for CTH-adjusted Levine age acceleration group-based trajectory analysis; **Table S7b.** Levine CTH-adjusted age acceleration trajectory group posterior model quality control evaluation; **Table S8a.** Model selection for Levine age acceleration group-based trajectory analysis; Table S8b. Levine age acceleration trajectory group posterior model quality control evaluation; **Table S9a.** Model selection for CTH-adjusted Levine age acceleration group-based trajectory analysis; **Table S9b.** Levine CTH-adjusted age acceleration trajectory group posterior model quality control evaluation.

Supplement2 **Additional file 2.** Probe IDs, clock coefficients, and availability of DNA methylation data for Horvath, Hannum, Levine, and Zhang epigenetic clocks.

## Figures and Tables

**Table 1:** Sample characteristics

Variable	Overall sample^a^ (*n* = 273)	Sample subset with both CSF and blood (*n* = 72)
Age, mean (*SD*)	52.9 (11.1)	53.0 (11.5)
Sex, female, *n* (%)	187 (68.5)	50 (69.4)
Race, White, *n* (%)	238 (87.2)	61 (84.7)
Fisher, *n* (%)		
*2*	81 (29.7)	20 (27.8)
*3*	135 (49.5)	37 (51.4)
*4*	57 (20.9)	15 (20.8)
Smoking, *n* (%)		
*No*	88 (32.2)	28 (38.8)
*Yes*	147 (53.8)	37 (51.4)
*Social*	3 (1.1)	2 (2.8)
*Quit*	31 (11.4)	3 (4.2)
*Unknown*	4 (1.5)	2 (2.8)
BMI, mean (*SD*)	28.1 (7.2)	28.8 (8.8)

*CSF*, cerebrospinal fluid; *SD*, standard deviation; *BMI*, body mass index

a All participants in this study had longitudinal CSF samples available over 14 days post-aSAH

**Table 2:** Summary of Horvath, Hannum, and Zhang cerebrospinal fluid DNA methylation age acceleration trajectory group characteristics

	Horvath (CSF)					Horvath (CSF) + CTH				
Variable	Group 1	Group 2	Group 3	Group 4	*p*	Group 1	Group 2	Group 3	Group 4	*p*
Group membership, n (%)	8 (2.9)	115 (42.1)	112 (41.0)	38 (13.9)		23 (8.4)	135 (49.5)	95 (34.8)	20 (7.3)	
Age, mean (SD)	48.5 (14.7)	53.2 (11.9)	53.0 (10.6)	52.5 (9.3)	0.863^b^	48.7 (11.6)	54.0 (11.8)	52.7 (10.0)	51.7 (9.5)	0.189^b^
DNAmAge, CSF, mean (SD)	46.5 (7.1)	55.1 (6.5)	58.5 (5.7)	62.0 (5.4)	**4.265E-13** ^b^	50.7 (6.5)	56.3 (6.5)	58.9 (5.8)	62.2 (6.1)	**1.647E-09** ^b^
Age Acceleration, CSF, mean (SD)	−8.3 (3.5)	−2.3 (1.3)	1.2 (1.1)	5.0 (1.7)	**<2.2E-16** ^b^	−4.3 (1.9)	−1.4 (2.3)	1.9 (1.7)	5.7 (1.9)	**<2.2E-16** ^b^
DNAmAge, Blood, mean (SD)	42.3 (9.2)	56.3 (8.6)	56.7 (7.0)	59.4 (5.1)	**0.047** ^b^	48.6 (7.0)	56.6 (7.9)	58.9 (6.8)	59.7 (3.2)	**6.509E-15** ^b^
Age Acceleration, Blood, mean (SD)	−1.1 (3.6)	−2.3 (3.8)	1.1 (2.3)	3.7 (1.7)	**2.935E-06** ^b^	−4.5 (2.8)	−1.2 (2.2)	2.3 (2.7)	4.6 (2.3)	**6.509E-15** ^b^
Sex, female, n (%)	6 (75.0)	87 (75.7)	71 (63.4)	23 (60.5)	0.15^c^	19 (82.6)	97 (71.9)	56 (58.9)	15 (75.0)	0.06^c^
Race, White, n (%)	6 (75.0)	105 (91.3)	95 (84.8)	32 (84.2)	0.30^c^	19 (82.6)	121 (89.6)	82 (86.3)	16 (80.0)	0.54^c^
Fisher										
*2*	1 (12.5)	28 (24.3)	41 (36.6)	11 (28.9)	0.36^c^	5 (21.7)	40 (29.6)	31 (32.6)	5 (25.0)	0.88^c^
*3*	6 (75.0)	59 (51.3)	51 (45.5)	19 (50.0)		12 (52.2)	65 (48.1)	46 (48.4)	12 (60.0)	
*4*	1 (12.5)	28 (24.3)	20 (17.9)	8 (21.1)		6 (26.1)	30 (22.2)	18 (18.9)	3 (15.0)	
Smoking history, n (%)										
*No*	2 (25.0)	38 (33.0)	35 (31.2)	13 (34.2)	0.95^c^	6 (26.1)	47 (34.8)	27 (28.4)	8 (40.0)	0.56^c^
*Yes* ^a^	6 (75.0)	76 (66.1)	75 (67.0)	24 (63.2)		16 (69.6)	87 (64.5)	67 (70.5)	11 (55.0)	
*Unknown*	0	1 (0.9)	2 (1.8)	1 (2.6)		1 (4.3)	1 (0.7)	1 (1.1)	1 (5.0)	
BMI, mean (SD)	26.5 (5.2)	29.3 (8.8)	27.1 (5.8)	27.8 (5.3)	0.145^b^	27.4 (6.8)	28.8 (7.9)	27.1 (6.3)	28.4 (6.6)	0.371^b^
	Hannum (CSF)					Hannum (CSF) + CTH				
Variable	Group 1	Group 2	Group 3	Group 4	*p*	Group 1	Group 2	Group 3	Group 4	*p*
Group membership, n (%)	15 (5.5)	121 (44.3)	131 (48.0)	6 (2.2)		10 (3.7)	102 (37.4)	124 (45.4)	37 (13.6)	
Age, mean (SD)	54.5 (9.9)	53.5 (11.2)	52.0 (11.3)	56.7 (7.3)	0.531^b^	53.5 (11.7)	53.6 (11.2)	51.6 (10.7)	55.0 (11.7)	0.333^b^
DNAmAge, CSF, mean (SD)	55.0 (5.9)	56.8 (6.4)	57.5 (7.1)	62.8 (3.8)	**4.809E-09** ^b^	55.7 (5.7)	56.9 (6.9)	56.7 (6.4)	60.1 (7.2)	**8.487E-07** ^b^
Age Acceleration, CSF, mean (SD)	−3.0 (3.5)	−0.7 (2.6)	0.8 (3.3)	3.6 (1.8)	**<2.2E-16** ^b^	−1.8 (4.1)	−0.7 (2.8)	0.1 (3.2)	1.8 (3.0)	**<2.2E-16** ^b^
DNAmAge, Blood, mean (SD)	54.4 (5.1)	57.5 (8.3)	55.7 (7.8)	53.0 (6.2)	**0.789** ^b^	53.5 (6.6)	55.4 (8.7)	57.1 (6.9)	60.1 (7.8)	**0.030** ^b^
Age Acceleration, Blood, mean (SD)	−0.7 (2.5)	−0.3 (4.0)	0.4 (3.3)	−0.4 (2.8)	**0.003** ^b^	−0.6 (2.6)	−1.7 (3.4)	1.4 (2.9)	2.5 (3.9)	**1.495E-06** ^b^
Sex, female, n (%)	14 (93.3)	100 (82.6)	72 (55.0)	1 (16.7)	**1.1E-7** ^c^	9 (90.0)	88 (86.3)	75 (60.5)	15 (40.5)	**1.1E-7** ^c^
Race, White, n (%)	11 (73.3)	104 (86.0)	118 (90.1)	5 (83.3)	0.28^c^	8 (80.0)	83 (81.4)	113 (91.1)	34 (91.9)	0.11^c^
Fisher										
*2*	5 (33.3)	34 (28.1)	42 (32.1)	1 (16.7)	0.62^c^	2 (20.0)	27 (26.5)	40 (32.3)	12 (32.4)	0.95^c^
*3*	8 (53.3)	58 (47.9)	66 (50.4)	3 (50.0)		6 (60.0)	52 (51.0)	59 (47.6)	18 (48.6)	
*4*	2 (13.3)	29 (24.0)	23 (17.6)	2 (33.3)		2 (20.0)	23 (22.5)	25 (20.2)	7 (18.9)	
Smoking history, n (%)										
*No*	3 (0.2)	39 (32.2)	44 (33.6)	2 (33.3)	0.75^c^	3 (0.3)	29 (28.4)	42 (33.9)	14 (37.8)	0.68^c^
*Yes* ^a^	12 (0.8)	79 (65.3)	85 (64.9)	4 (66.7)		6 (0.6)	73 (71.6)	79 (63.7)	23 (62.2)	
*Unknown*	0	3 (2.5)	2 (1.5)	0		1 (0.1)	0	3 (2.4)	0	
BMI, mean (SD)	26.3 (8.7)	28.1 (7.6)	28.5 (6.8)	26.8 (1.8)	0.720^b^	25.4 (3.0)	28.3 (8.5)	28.1 (6.6)	28.6 (6.0)	0.698^b^
	Zhang (CSF)									
Variable	Group 1	Group 2	Group 3	Group 4	*p*					
Group membership, n (%)	47 (17.2)	148 (54.2)	71 (26.0)	7 (2.6)						
Age, mean (SD)	52.2 (14.1)	53.3 (10.8)	52.6 (9.6)	52.3 (10.5)	0.940^b^					
DNAmAge, CSF, mean (SD)	50.3 (11.5)	53.9 (9.0)	56.2 (8.1)	59.3 (8.7)	**0.004** ^b^					
Age Acceleration, CSF, mean (SD)	−3.1 (0.9)	−0.4 (0.9)	2.4 (0.9)	5.8 (1.8)	**<2.2E-16** ^b^					
DNAmAge, Blood, mean (SD)	47.6 (12.4)	55.7 (10.2)	56.1 (7.5)	52.1 (4.0)	**0.063** ^b^					
Age Acceleration, Blood, mean (SD)	−2.8 (1.1)	−0.2 (1.7)	2.3 (1.3)	2.4 (4.7)	**2.583E-12** ^b^					
Sex, female, n (%)	37 (78.7)	98 (66.2)	49 (69.0)	3 (42.9)	0.19^c^					
Race, White, n (%)	40 (85.1)	127 (85.8)	64 (90.1)	6 (85.7)	**1.1E-7** ^c^					
Fisher										
*2*	11 (23.4)	45 (30.4)	24 (33.8)	1 (14.3)	0.76^c^					
*3*	26 (55.3)	73 (49.3)	31 (43.7)	5 (71.4)						
*4*	10 (21.3)	30 (20.3)	16 (22.5)	1 (14.3)						
Smoking history, n (%)										
*No*	14 (29.8)	49 (33.1)	23 (32.4)	2 (28.6)	0.97^c^					
*Yes* ^a^	32 (68.1)	96 (64.9)	48 (67.6)	5 (71.4)						
*Unknown*	1 (2.1)	3 (2.0)	0	0						
BMI, mean (SD)	29.8 (10.5)	27.3 (6.1)	28.1 (5.6)	33.0 (12.2)	0.067^b^					

The table corresponds to Fig. 6; Zhang + CTH is not included here because the trajectory model did not pass QC

*CTH*, cell-type heterogeneity; *SD*, standard deviation; *CSF*, cerebrospinal fluid; BMI, body mass index

a Smoking history: yes (current, social, and past smokers), no (never smoked)

b One-way ANOVA

c Chi-square or Fisher’s exact

**Table 3:** Bivariate associations between participant characteristics and DNA methylation age acceleration metrics independent of trajectory group

Age acceleration metric	Sex			Self-reported race		
	Est (SE)	95% CI	*p*	Est (SE)	95% CI	*p*
Horvath (CSF)	− 0.96 (0.41)	− 1.78 to − 0.15	**0.02** ^a^	0.31 (0.58)	− 0.84 to 1.45	0.60
Horvath (CSF) + CTH	− 0.63 (0.50)	− 1.62 to 0.37	0.22	1.10 (0.67)	− 0.22 to 2.42	0.10
Horvath (blood)	− 0.24 (0.93)	− 2.09 to 1.61	0.80	− 0.36 (1.23)	− 2.82 to 2.10	0.77
Horvath (blood) + CTH	− 0.30 (0.73)	− 1.76 to 1.15	0.96	0.34 (0.97)	− 1.60 to 2.27	0.73
Hannum (CSF)	− 2.78 (0.44)	− 3.65 to − 1.92	**8.75E−10** ^a^	− 0.92 (0.65)	− 2.20 to 0.36	0.16
Hannum (CSF) + CTH	− 2.18 (0.55)	− 3.27 to − 1.10	**0.0001** ^a^	− 0.06 (0.77)	− 1.58 to 1.46	0.94
Hannum (blood)	− 0.69 (0.80)	− 2.29 to 0.91	0.39	− 2.23 (1.04)	− 4.30 to − 0.15	**0.04** ^a^
Hannum (Blood) + CTH	− 0.53 (0.58)	− 1.69 to 0.63	0.37	− 0.21 (0.78)	− 1.76 to 1.35	0.79
Levine (CSF)	− 0.30 (0.61)	− 1.50 to 0.90	0.62	1.81 (0.84)	0.84 to 2.22	**0.03** ^a^
Levine (CSF) + CTH	0.99 (0.75)	− 0.49 to 2.47	0.19	2.94 (0.97)	1.02 to 4.87	**0.003** ^a^
Levine (blood)	− 0.11 (1.05)	− 2.2 to 1.99	0.92	− 1.16 (1.39)	− 3.93 to 1.62	0.41
Levine (blood) + CTH	0.12 (0.95)	− 1.77 to 2.01	0.90	0.17 (1.26)	− 2.34 to 2.69	0.89
Zhang (CSF)	− 0.31 (0.29)	− 0.88 to 0.26	0.29	− 0.58 (0.40)	− 1.37 to 0.21	0.15
Zhang (CSF) + CTH	− 0.09 (0.44)	− 0.95 to 0.78	0.85	0.04 (0.58)	− 1.11 to 1.19	0.95
Zhang (blood)	0.74 (0.60)	− 0.45 to 1.93	0.22	− 0.25 (0.80)	− 1.85 to 1.35	0.75
Zhang (blood) + CTH	0.73 (0.53)	− 0.33 to 1.79	0.17	0.43 (0.72)	− 1.00 to 1.85	0.55
Age acceleration metric	Smoking history			BMI		
	Est (SE)	95% CI	*p*	Est (SE)	95% CI	*p*
Horvath (CSF)	− 0.07 (0.41)	− 0.89 to 0.74	0.86	− 0.18 (0.14)	− 0.46 to 0.10	0.22
Horvath (CSF) + CTH	0.24 (0.49)	− 0.74 to 1.21	0.63	0.04 (0.24)	− 0.42 to 0.51	0.86
Horvath (blood)	− 0.86 (0.88)	− 2.62 to 0.90	0.33	0.10 (0.31)	− 0.51 to 0.71	0.74
Horvath (blood) + CTH	− 0.83 (0.68)	− 2.19 to 0.54	0.23	0.24 (0.38)	− 0.52 to 0.99	0.53
Hannum (CSF)	− 0.57 (0.47)	− 1.49 to 0.35	0.22	0.08 (0.13)	− 0.17 to 0.33	0.52
Hannum (CSF) + CTH	0.17 (0.56)	− 0.94 to 1.27	0.77	0.19 (0.21)	− 0.21 to 0.60	0.35
Hannum (blood)	− 1.11 (0.76)	− 2.62 to 0.40	0.15	0.14 (0.35)	− 0.55 to 0.83	0.69
Hannum (blood) + CTH	− 0.23 (0.56)	− 1.34 to 0.89	0.69	0.02 (0.03)	− 0.05 to 0.08	0.61
Levine (CSF)	0.84 (0.61)	− 0.36 to 2.04	0.17	0.01 (0.09)	− 0.18 to 0.20	0.89
Levine (CSF) + CTH	2.09 (0.70)	0.70 to 3.48	**0.003** ^a^	− 0.09 (0.16)	− 0.40 to 0.22	0.56
Levine (blood)	− 0.06 (0.97)	− 2.05 to 1.93	0.96	0.18 (0.26)	− 0.35 to 0.70	0.51
Levine (blood) + CTH	0.44 (0.90)	− 1.34 to 2.23	0.62	0.03 (0.05)	− 0.07 to 0.13	0.55
Zhang (CSF)	− 0.17 (0.29)	− 0.74 to 0.41	0.57	− 0.04 (0.20)	− 0.43 to 0.36	0.85
Zhang (CSF) + CTH	0.46 (0.42)	− 0.38 to 1.29	0.28	− 0.01 (0.26)	− 0.53 to 0.52	0.98
Zhang (blood)	− 0.33 (0.57)	− 1.47 to 0.82	0.57	− 0.42 (0.46)	− 1.33 to 0.50	0.37
Zhang (blood) + CTH	− 0.31 (0.51)	− 1.33 to 0.72	0.56	− 0.03 (0.03)	− 0.09 to 0.03	0.32

The results of age acceleration metrics regressed on participant characteristics

*Est (SE)*, unstandardized estimate and standard error; *CI*, confidence interval

a Significance based on an alpha of 0.05; sex, reference = male; self-reported race, reference = White; smoking history, reference = no

## References

[1] Lantigua H, Ortega-Gutierrez S, Schmidt JM, Lee K, Badjatia N, Agarwal S, Subarachnoid hemorrhage: who dies, and why? Crit Care 2015;19(1): 309. 10.1186/s13054-015-1036-0. 26330064 PMC4556224

[2] Horvath S, Raj K. DNA methylation-based biomarkers and the epigenetic clock theory of ageing. Nature Reviews Genetics 2018;19(6):371–84. 10.1038/s41576-018-0004-3 29643443

[3] Horvath S DNA methylation age of human tissues and cell types. Genome Biol 2013;14(10):R115. 10.1186/gb-2013-14-10-r115. 24138928 PMC4015143

[4] Horvath S Erratum to: DNA methylation age of human tissues and cell types. Genome Biol 2015;16(1):96. 10.1186/s13059-015-0649-6. 25968125 PMC4427927

[5] Hannum G, Guinney J, Zhao L, Zhang L, Hughes G, Sadda SV, Genome-wide methylation profiles reveal quantitative views of human aging rates. Mol Cell 2013;49(2):359–67. 10.1016/j.molcel.2012.10.016. 23177740 PMC3780611

[6] Levine ME, Lu AT, Quach A, Chen BH, Assimes TL, Bandinelli S, An epigenetic biomarker of aging for lifespan and healthspan. Aging (Albany NY) 2018;10(4):573–91. 10.18632/aging.101414. 29676998 PMC5940111

[7] Zhang Q, Vallerga CL, Walker RM, Lin T, Henders AK, Montgomery GW, Improved precision of epigenetic clock estimates across tissues and its implication for biological ageing. Genome Med 2019;11(1):54. 10.1186/s13073-019-0667-1. 31443728 PMC6708158

[8] Kresovich JK, Xu Z, O’Brien KM, Weinberg CR, Sandler DP, Taylor JA. Methylation-based biological age and breast cancer risk. J Natl Cancer Inst 2019;111(10):1051–8. 10.1093/jnci/djz020. 30794318 PMC6792078

[9] Horvath S, Ritz BR. Increased epigenetic age and granulocyte counts in the blood of Parkinson’s disease patients. Aging (Albany NY) 2015;7(12):1130–42. 10.18632/aging.100859. 26655927 PMC4712337

[10] Roetker NS, Pankow JS, Bressler J, Morrison AC, Boerwinkle E. Prospective study of epigenetic age acceleration and incidence of cardiovascular disease outcomes in the ARIC Study (atherosclerosis risk in communities). Circ Genomic Precis Med 2018;11(3):e001937. 10.1161/CIRCGEN.117.001937. PMC5863591 29555670

[11] Marioni RE, Shah S, McRae AF, DNA methylation age of blood predicts all-cause mortality in later life. Genome Biol 2015;16(1):25. 10.1186/s13059-015-0584-6. 25633388 PMC4350614

[12] Bell CG, Lowe R, Adams PD, Baccarelli AA, Beck S, Bell JT, DNA methylation aging clocks: challenges and recommendations. Genome Biol 2019;20(1):249. 10.1186/s13059-019-1824-y. 31767039 PMC6876109

[13] Sakka L, Coll G, Chazal J. Anatomy and physiology of cerebrospinal fluid. Eur Ann Otorhinolaryngol Head Neck Dis 2011;128(6):309–16. 10.1016/j.anorl.2011.03.002. 22100360

[14] van Gijn J, Kerr RS, Rinkel GJE. Subarachnoid haemorrhage. Lancet 2007; 369(9558):306–18. 10.1016/S0140-6736(07)60153-6. 17258671

[15] Wang X, Mori T, Sumii T, Lo EH. Hemoglobin-induced cytotoxicity in rat cerebral cortical neurons: caspase activation and oxidative stress. Stroke 2002;33(7):1882–8. 10.1161/01.STR.0000020121.41527.5D. 12105370

[16] Yang Y, Chen S, Zhang J-M. The updated role of oxidative stress in subarachnoid hemorrhage. Curr Drug Deliv 2017;14(6):832–42. 10.2174/1567201813666161025115531. 27784210

[17] Rang FJ, Boonstra J. Causes and consequences of age-related changes in DNA methylation: a role for ROS? Biology (Basel) 2014;3(2):403–25. 10.3390/biology3020403. 24945102 PMC4085615

[18] Armstrong MJ, Jin Y, Allen EG, Jin P. Diverse and dynamic DNA modifications in brain and diseases. Hum Mol Genet 2019;28(R2):R241–53. 10.1093/hmg/ddz179. 31348493 PMC6872432

[19] Lappalainen T, Greally JM. Associating cellular epigenetic models with human phenotypes. Nat Rev Genet 2017;18(7):441–51. 10.1038/nrg.2017.32. 28555657

[20] de Reuck J, Vanderdonckt P. Choroid plexus and ependymal cells in CSF cytology. Clin Neurol Neurosurg 1986;88(3):177–9. 10.1016/S0303-8467(86)80025-7. 3780107

[21] McGregor K, Bernatsky S, Colmegna I, Hudson M, Pastinen T, Labbe A, An evaluation of methods correcting for cell-type heterogeneity in DNA methylation studies. Genome Biol 2016;17(1):84. 10.1186/s13059-016-0935-y. 27142380 PMC4855979

[22] Zacharia BE, Hickman ZL, Grobelny BT, DeRosa P, Kotchetkov I, Ducruet AF, Epidemiology of aneurysmal subarachnoid hemorrhage. Neurosurg Clin N Am 2010;21(2):221–33. 10.1016/j.nec.2009.10.002. 20380965

[23] El Khoury LY, Gorrie-Stone T, Smart M, Systematic underestimation of the epigenetic clock and age acceleration in older subjects. Genome Biol 2019;20(1):283. 10.1186/s13059-019-1810-4. 31847916 PMC6915902

[24] Houseman EA, Molitor J, Marsit CJ. Reference-free cell mixture adjustments in analysis of DNA methylation data. Bioinformatics 2014;30(10):1431–9. 10.1093/bioinformatics/btu029. 24451622 PMC4016702

[25] Teschendorff AE, Zhu T, Breeze CE, Beck S. EPISCORE: cell type deconvolution of bulk tissue DNA methylomes from single-cell RNA-Seq data. Genome Biol 2020;21(1):221. 10.1186/s13059-020-02126-9. 32883324 PMC7650528

[26] Horvath S, Gurven M, Levine ME, Trumble BC, Kaplan H, Allayee H, An epigenetic clock analysis of race/ethnicity, sex, and coronary heart disease. Genome Biol 2016;17(1):171. 10.1186/s13059-016-1030-0. 27511193 PMC4980791

[27] Mayne BT, Leemaqz SY, Smith AK, Breen J, Roberts CT, Bianco-Miotto T. Accelerated placental aging in early onset preeclampsia pregnancies identified by DNA methylation. Epigenomics 2017;9(3):279–89. 10.2217/epi-2016-0103. 27894195 PMC6040051

[28] Coninx E, Chew YC, Yang X, Guo W, Coolkens A, Baatout S, Hippocampal and cortical tissue-specific epigenetic clocks indicate an increased epigenetic age in a mouse model for Alzheimer’s disease. Aging (Albany NY) 2020;12(20):20817–34. 10.18632/aging.104056. 33082299 PMC7655172

[29] Gujarati D, Porter D. Basic econometrics 5th ed. McGraw-Hill Publishing Company, 2009.

[30] Arockiaraj AI, Liu D, Shaffer JR, Methylation data processing protocol and comparison of blood and cerebral spinal fluid following aneurysmal subarachnoid hemorrhage. Front Genet Epub ahead of print 26 June 2020. DOI: 10.3389/fgene.2020.00671. PMC7332758 32670358

[31] Li S, Wong EM, Bui M, Inference about causation between body mass index and DNA methylation in blood from a twin family study. International Journal of Obesity November 2018;21:1–10. 10.1038/s41366-018-0103-4 29777239

[32] Li S, Wong EM, Bui M, Nguyen TL, Joo JHE, Stone J, Causal effect of smoking on DNA methylation in peripheral blood: a twin and family study. Clin Epigenetics 2018;10(1):18. 10.1186/s13148-018-0452-9. 29456763 PMC5810186

[33] Fisher CM, Kistler JP, Davis JM. Relation of cerebral vasospasm to subarachnoid hemorrhage visualized by computerized tomographic scanning. Neurosurgery 1980;6(1):1–9. 10.1227/00006123-198001000-00001. 7354892

[34] Miller SA, Dykes DD, Polesky HF. A simple salting out procedure for extracting DNA from human nucleated cells. Nucleic Acids Res 1988;16(3): 1215. 10.1093/nar/16.3.1215. 3344216 PMC334765

[35] Oros Klein K, Grinek S, Bernatsky S, Bouchard L, Ciampi A, Colmegna I, FuntooNorm: an R package for normalization of DNA methylation data when there are multiple cell or tissue types. Bioinformatics 2016;32(4):593–5. 10.1093/bioinformatics/btv615. 26500152 PMC4743629

[36] Gervin K, Page CM, Aass HCD, Jansen MA, Fjeldstad HE, Andreassen BK, Cell type specific DNA methylation in cord blood: a 450 K-reference data set and cell count-based validation of estimated cell type composition. Epigenetics 2016;11(9):690–8. 10.1080/15592294.2016.1214782. 27494297 PMC5048717

[37] Sidiropoulos N, Sohi SH, Pedersen TL, Porse BT, Winther O, Rapin N, SinaPlot: an enhanced chart for simple and truthful representation of single observations over multiple classes. J Comput Graph Stat 2018;27(3):673–6. 10.1080/10618600.2017.1366914.

[38] Pidsley RY Wong CC, Volta M, A data-driven approach to preprocessing Illumina 450 K methylation array data. BMC Genomics 2013; 14(1):293. 10.1186/1471-2164-14-293. 23631413 PMC3769145

[39] Team RC. R: a language and environment for statistical computing, https://www.r-project.org/ (2018).

[40] Zhang Q DNA methylation based chronological age predictor Epub ahead of print. 2019. 10.5281/zenodo.3369456.

[41] Jones BL, Nagin DS, Roeder K. A SAS procedure based on mixture models for estimating developmental trajectories. Sociol Methods Res 2001;29(3): 374–93. 10.1177/0049124101029003005.

[42] Jones BL, Nagin DS. Advances in group-based trajectory modeling and an SAS procedure for estimating them. Sociol Methods Res 2007;35(4):542–71. 10.1177/0049124106292364.

