# Supplementary material for: Characterization of cerebrospinal fluid DNA methylation age during the acute recovery period following aneurysmal subarachnoid hemorrhage
Source: Epigenetics Commun. Author manuscript; Available in PMC 2022 Jan 25. (PMC8787331; doi:10.1186/s43682-021-00002-6)
Supplement: Supplement1 — Additional file 1: Supplementary Methods. DNAm age calculation; Table S1. Summary of missing probes in CSF and blood for Horvath, Hannum, Levine, and Zhang epigenetic clocks; Figure S1. Regression line overlays comparing chronological age versus DNAm age in CSF over 14 days post-aSAH using the Horvath, Hannum, Levine, and Zhang epigenetic clocks; Figure S2. Chronological age versus DNA methylation age in CSF at cross-sectional time points over 14 days post-aSAH using the Horvath epigenetic clock; Figure S3. Chronological age versus DNAm age in CSF at cross-sectional time points over 14 days post-aSAH using the Hannum epigenetic clock; Figure S4. Chronological age versus DNAm age in CSF at cross-sectional time points over 14 days post-aSAH using the Levine epigenetic clock; Figure S5. Chronological age versus DNAm age in CSF at cross-sectional time points over 14 days post-aSAH using the Zhang epigenetic clock; Figure S6. Regression line overlays comparing chronological age versus DNAm age in blood at Time 1 (Days 0 to 2) post-aSAH using the Horvath, Hannum, Levine, and Zhang epigenetic clocks; Figure S7. Correlation heatmap of DNA methylation age at Time 1 (days 0 to 2) post-aSAH computed in CSF and blood using the Horvath, Hannum, Levine, and Zhang epigenetic clocks; Figure S8. Sina with violin plots of CSF putative cell types included in our CTH-adjusted analyses; Figure S9. Spaghetti plots of CSF putative cell types included in our CTH-adjusted analyses; Figure S10. Trajectory plots of unadjusted and CTH-adjusted CSF age acceleration from Levine and Zhang epigenetic clocks; Table S2a. Model selection for Horvath age acceleration group-based trajectory analysis; Table S2b. Horvath age acceleration trajectory group posterior model quality control evaluation; Table S3a. Model selection for CTH-adjusted Horvath age acceleration group-based trajectory analysis; Table S3b. Horvath CTH-adjusted age acceleration trajectory group posterior model quality control evaluation [file NIHMS1770582-supplement-Supplement1.docx]

**Characterization of Cerebrospinal Fluid DNA Methylation Age During the Acute**

**Recovery Period Following Aneurysmal Subarachnoid Hemorrhage**

Supplementary Material, Additional File 1

Lacey W. Heinsberg, PhD, RN*; Dongjing Liu, PhD; John R. Shaffer, PhD;

Daniel E. Weeks, PhD; Yvette P. Conley, PhD, FAAN

*Corresponding author

Department of Human Genetics

Graduate School of Public Health

University of Pittsburgh

E-mail: law145@pitt.edu

# Supplementary Methods

## DNA Methylation Age

DNAm age was calculated using three epigenetic clocks common in the literature (Horvath^1,2^, Hannum^3^, and Levine^4^). These methods use linear functions and clock-specific probes and coefficients to compute DNAm age as shown in Equation 1:

$DNAmAge={m_{0}+ m}_{1}\beta_{1}+m_{2}\beta_{2}+\ldots+ m_{n}\beta_{n}$ (1)

Where $DNAmAge$ is the predicted DNA methylation age for a given individual, $m$ is a clock-specific coefficient corresponding to a clock-specific probe, $\beta$ is the DNA methylation measurement, , a beta value as measured on a 0 to 1 scale, for a clock-specific probe within a given individual, and $m_{0}$ is a clock-specific model intercept. It should be noted that the Horvath method also uses an age transformation function as described elsewhere and shown in the R code below.^1,2^

DNAm age was computed for the Hannum, Horvath, and Levine epigenetic clocks using a modified function from the wateRmelon package^5^ in R^6^ (wateRmelon:agep). The wateRmelon package supplies both Horvath and Hannum coefficients for use with the ‘agep’ function and we modified this function to also compute Levine DNAm age as shown below. The modified function used to compute DNAm data can be found below. DNAm age was computed for the Zhang ‘Improved Precision’ Clock ^7^using the Elastic Net method from publicly available source code.^8^ Note that we were missing DNA methylation data for the cocks as summarized in Supplementary Table 1 below and detailed in the Supplementary excel file provided with this paper.

# Load Hannum coefficients (available through wateRmelon package)

data("hannumCoef")

hannum.coef <- hannumCoef

hannum.coef

# Read in Levine coefficients (located as a supplement to the original Levine paper

# An epigenetic biomarker of aging for lifespan and healthspan

# Levine, 2018)

levine <- read.csv("levine.csv", header=F)

levine.coef <- deframe(levine)

levine.coef

# Functions for Horvath, Hannum, and Levine epigenetic clocks

# Initial version: 20 Oct 2015 Leo

# Updated version: 18 Jul 2016 TGS

# Updated once more June 2018 TGS

# Updated Feb 2020 LWH to add Levine's clock

# Horvath's age transformation functions

trafo <- function(x,adult.age=20) { x=(x+1)/(1+adult.age); y=ifelse(x<=1, log( x),x-1);y }

anti.trafo <- function(x,adult.age=20) { ifelse(x<0, (1+adult.age)*exp(x)-1, (1+adult.age)*x+adult.age) }

# 'agep' function from wateRmelon package modified to 'LWH_agep' function Feb 2020

# to compute Levine DNAmAge in addition to Horvath and Hannum

LWH_agep <- function(betas, coeff = NULL, method = c('horvath', 'hannum', 'levine'),...){ # LWH: Open bracket

method <- match.arg(method)

# LWH: If coeff argument not provided, default to Horvath's clock

if(method == 'hannum' & is.null(coeff)) stop('Please supply coeffs for hannum\'s clock.')

if(method == 'levine' & is.null(coeff)) stop('Please supply coeffs for levine\'s clock.')

if(is.null(coeff)){

message('No coefficients detected, using Horvaths clock')

data(coef)

coeff <- coef

}

if(method == 'horvath'){

ages <- as.matrix(apply(betas,2,function(x){

# LWH: Determine number of CpGs missing from clock and omit

miss <- names(coeff)[-1]%in%names(na.omit(x))

coef2 <- coeff[-1][miss]

data <- x[names(coef2)]

# Compute values using DNA methylation and Horvath's coefficients

pre <- data %*% coef2 + coeff[1] # 'coeff[1] is Horvath intercept (0.6955)

# Use Horvath age function transformation to compute DNAmAge

anti.trafo(pre, adult.age=20)

}))

}

else if(method == 'hannum'){

ages <- as.matrix(apply(betas,2,function(x){

# LWH: Determine number of CpGs missing from clock and omit

miss <- names(coeff)%in%names(na.omit(x))

coef2 <- coeff[miss]

data <- x[names(coef2)]

# LWH: Multiply methylation by Hannum coefficients, compute sum for DNAmAge

data %*% coef2 + 0 # LWH: No model intercept for Hannum

}))

}

# Newly added by LWH

if (method == 'levine'){

ages <- as.matrix(apply(betas,2,function(x){

# Determine number of CpGs missing

miss <- names(coeff)%in%names(na.omit(x))

coef2 <- coeff[miss]

data <- x[names(coef2)]

# Multiply methylation by Levine coefficients, compute sum and add model intercept for DNAmAge

data %*% coef2 + 60.664 # Levine model intercept

}))

}

# LWH: Returns DNAmAge

return(ages)

} # LWH: Closing bracket

# Compute DNAmAge Horvath

horvath <- LWH_agep(beta, method=c('horvath')) # Horvath

# Compute DNAmAge Hannum

hannum <- LWH_agep(beta, coeff = hannum.coef, method=c('hannum'))

# Compute DNAmAge Levine

levine <- LWH_agep(beta, coeff = levine.coef, method=c('levine'))

# Supplementary Results

Supplementary figures and tables have been provided below.

**Table S1.** Summary of missing probes in CSF and blood for Horvath, Hannum, and Levine epigenetic clocks

| **Epigenetic Clock** | **Total number of probes** | **Number of missing probes in CSF data** | **Number of missing probes in blood data** | **Total number of probes removed due to missingness in either CSF or blood** |
| --- | --- | --- | --- | --- |
| Horvath | 353 | 1 | 1 | 1 |
| Hannum | 71 | 3 | 3 | 3 |
| Levine | 513 | 4 | 5 | 5 |
| Zhang | 514 | 11 | 11 | 11 |

Probe IDs, clock coefficients, and availability of data for this study are presented in detail in the Supplementary excel file included with this paper

**Figure S1.** Regression line overlays comparing chronological age versus DNAm age in CSF post-aSAH using the Horvath, Hannum, Levine, and Zhang epigenetic clocks


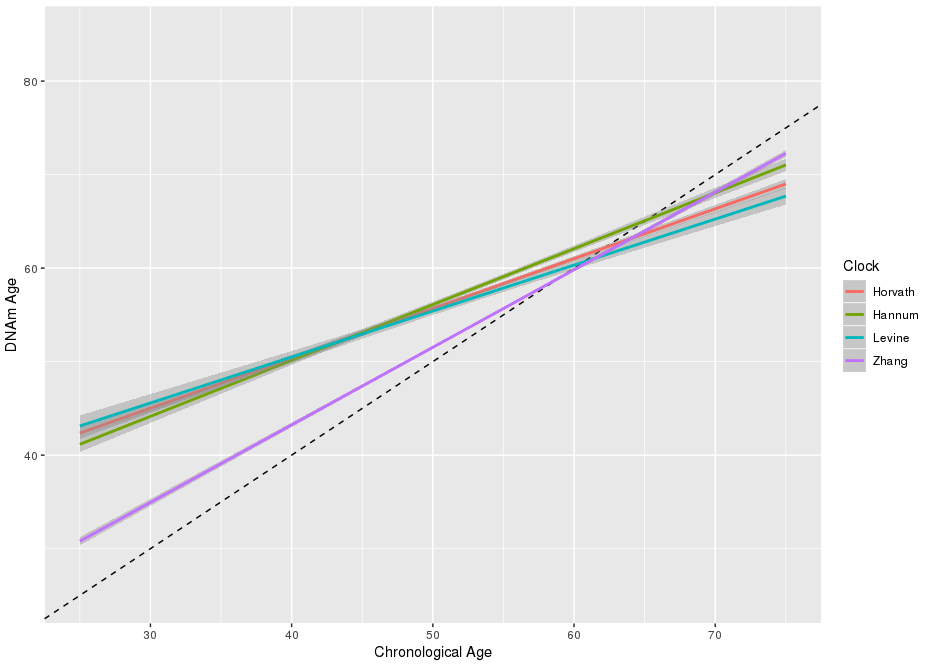


Overlay of fitted regression lines from Figure 1 in main text; n=273 at up to 5 time points (N=850 observations over 14 days post-aSAH); dashed line, y=x; solid line, predicted model fit; DNAm, DNA methylation; CSF, cerebrospinal fluid; aSAH, aneurysmal subarachnoid hemorrhage

**Figure S2.** Chronological age versus DNAm age in CSF at cross-sectional time points over 14 days post-aSAH using the Horvath epigenetic clock

**
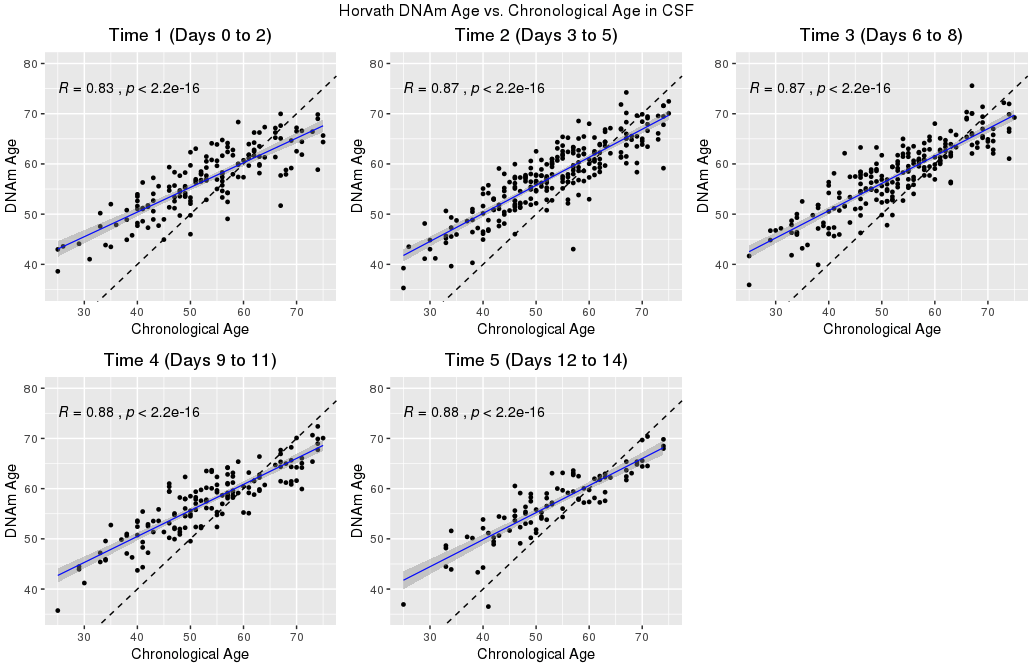
**

CSF, cerebrospinal fluid; DNAm, DNA methylation; aSAH, aneurysmal subarachnoid hemorrhage; *R*, correlation computed using Pearson method; dashed line, y=x; solid line, predicted model fit; regression of *R*~time (*p*=0.08)

**Figure S3.** Chronological age versus DNAm age in CSF at cross-sectional time points over 14 days post-aSAH using the Hannum epigenetic clock


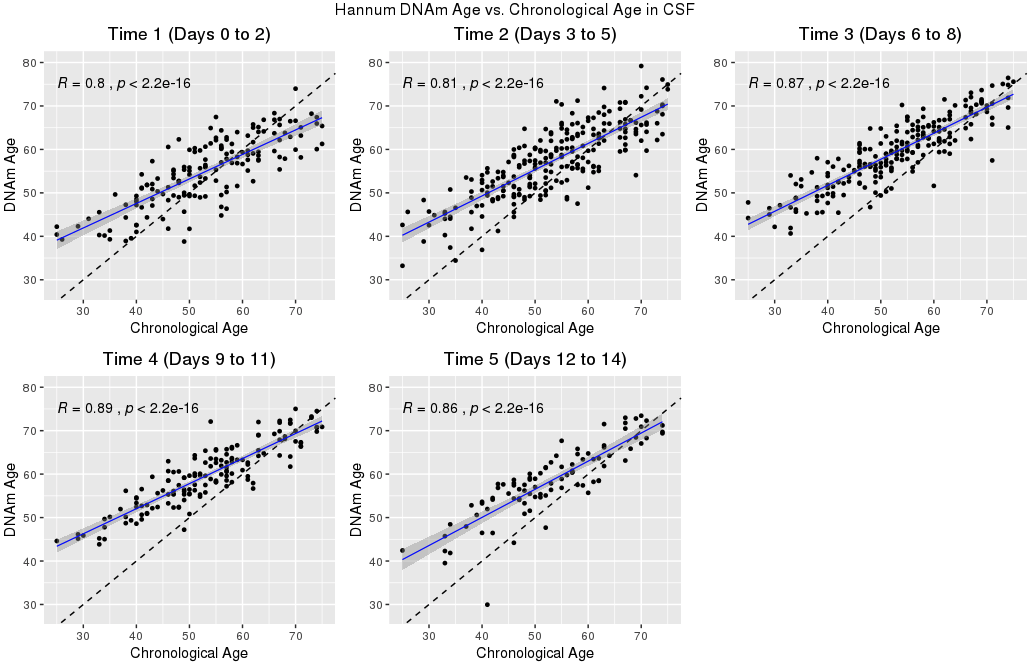


CSF, cerebrospinal fluid; DNAm, DNA methylation; aSAH, aneurysmal subarachnoid hemorrhage; *R*, correlation computed using Pearson method; dashed line, y=x; solid line, predicted model fit; regression of *R*~time (*p*=0.10)

**Figure S4.** Chronological age versus DNAm age in CSF at cross-sectional time points over 14 days post-aSAH using the Levine epigenetic clock


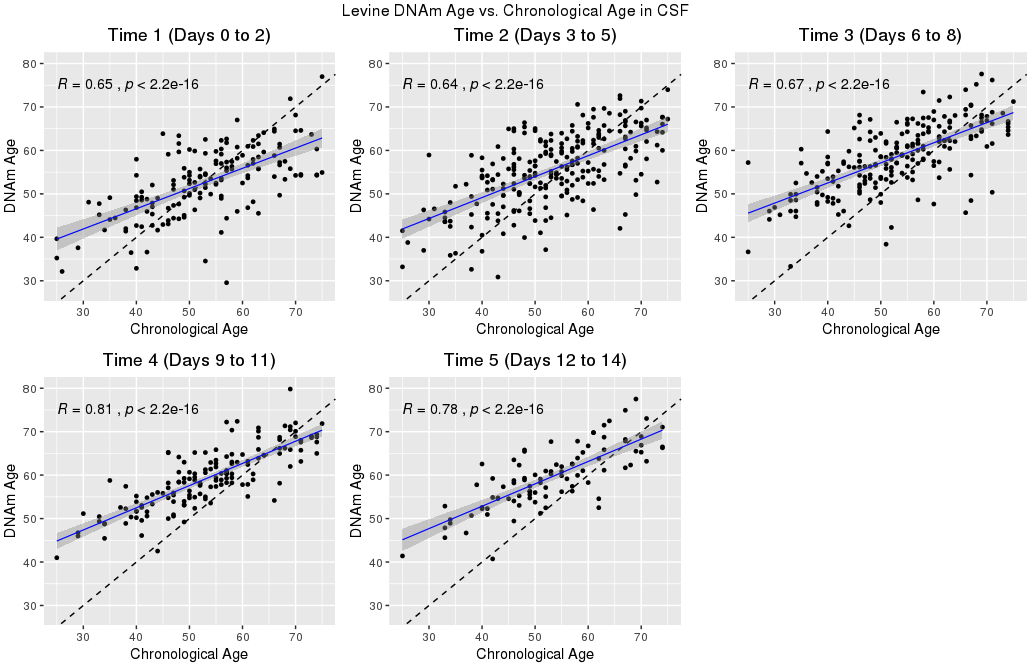


CSF, cerebrospinal fluid; DNAm, DNA methylation; aSAH, aneurysmal subarachnoid hemorrhage; *R*, correlation computed using Pearson method; dashed line, y=x; solid line, predicted model fit; regression of *R*~time (*p*=0.06)

**Figure S5.** Chronological age versus DNAm age in CSF at cross-sectional time points over 14 days post-aSAH using the Zhang epigenetic clock

**
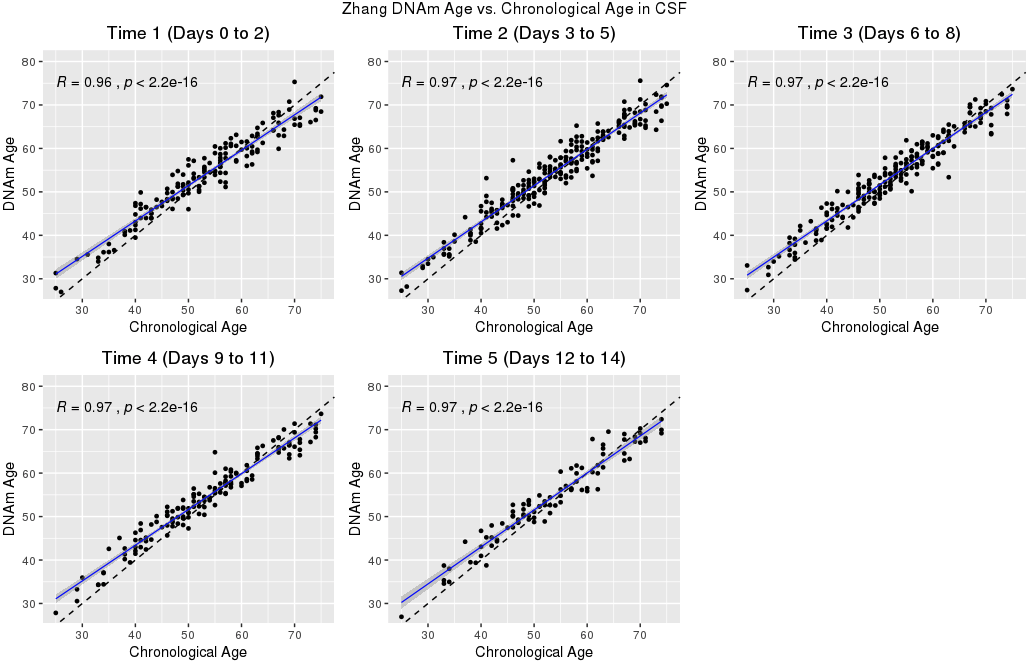
**

CSF, cerebrospinal fluid; DNAm, DNA methylation; aSAH, aneurysmal subarachnoid hemorrhage; *R*, correlation computed using Pearson method; dashed line, y=x; solid line, predicted model fit; regression of *R*~time (*p*=0.18)

**Figure S6.** Regression line overlays comparing chronological age versus DNAm age in blood post-aSAH using the Horvath, Hannum, Levine, and Zhang epigenetic clocks

**
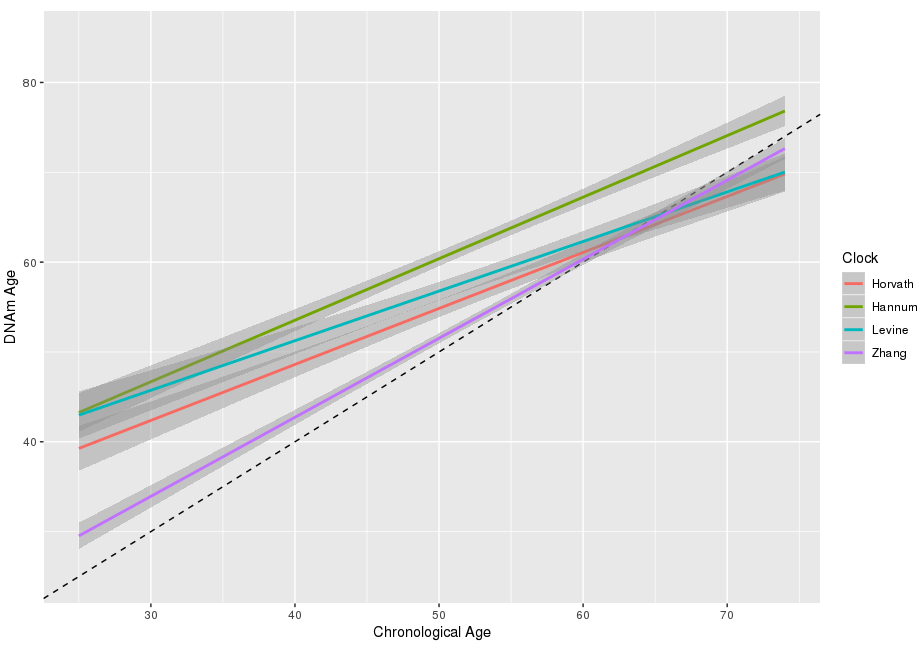
**

Overlay of fitted regression lines from Figure 2 in main text; n=72 at cross-sectional time point 1 (days 0 to 2 post-aSAH); dashed line, y=x; solid line, predicted model fit; DNAm, DNA methylation; aSAH, aneurysmal subarachnoid hemorrhage

**Figure S7.** Correlation heatmap of DNA methylation age at Time 1 (days 0 to 2) post-aSAH computed in CSF and blood using the Horvath, Hannum, Levine, and Zhang epigenetic clocks

**
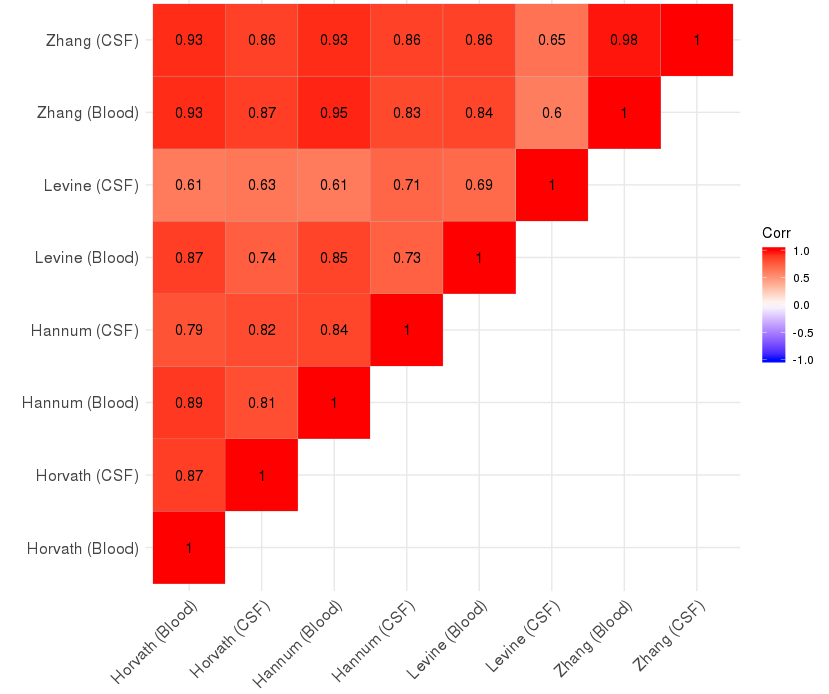
**

Sample size, n=72 with both CSF and blood DNA methylation data at cross-sectional time point 1 (days 0 to 2 post-aSAH); CSF, cerebrospinal fluid; aSAH, aneurysmal subarachnoid hemorrhage; all values presented are *R* values indicating age acceleration correlation computed using Pearson method

**Figure S8.** Sina with violin plots of CSF putative cell types included in our CTH-adjusted analyses **
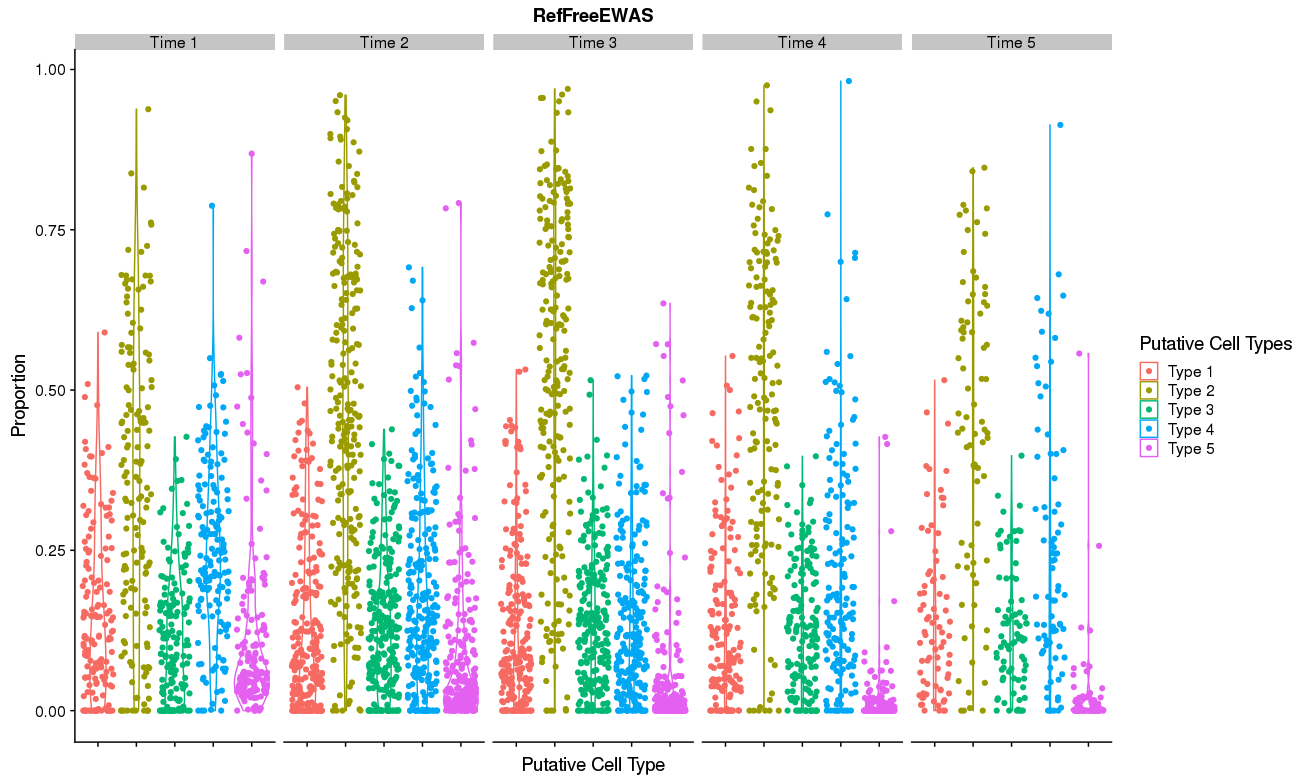
**

CSF, cerebrospinal fluid; CTH, cell type heterogeneity; Time 1 (days 0 to 2 post-aSAH); Time 2 (days 3 to 5 post-aSAH); Time 3 (days 6 to 8 post-aSAH); Time 4 (days 9 to 11 post-aSAH); Time 1 (days 12 to 14 post-aSAH); aSAH, aneurysmal subarachnoid hemorrhage

**Figure S9.** Spaghetti plots of CSF putative cell types included in our CTH-adjusted analyses

**
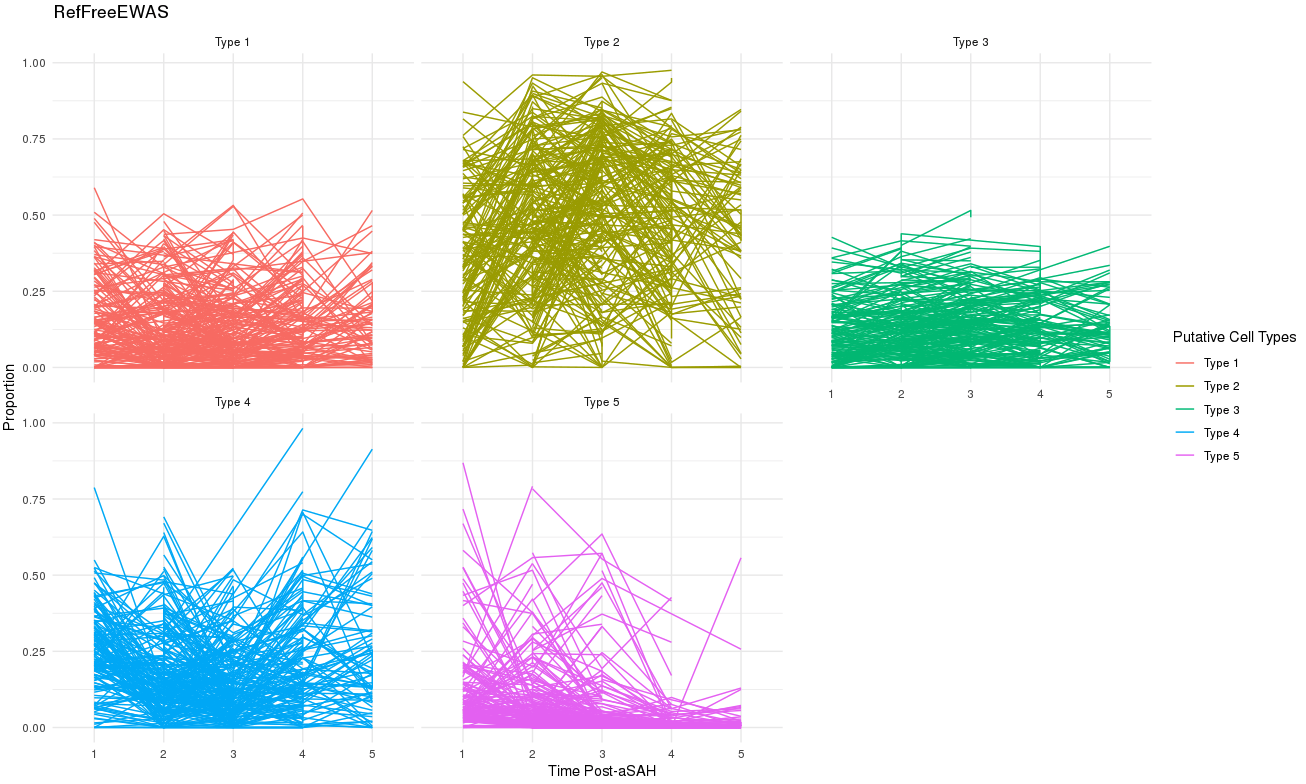
**

CSF, cerebrospinal fluid; CTH, cell type heterogeneity; Time 1 (days 0 to 2 post-aSAH); Time 2 (days 3 to 5 post-aSAH); Time 3 (days 6 to 8 post-aSAH); Time 4 (days 9 to 11 post-aSAH); Time 1 (days 12 to 14 post-aSAH); aSAH, aneurysmal subarachnoid hemorrhage

**Figure S10**. Trajectory plots of unadjusted and CTH-adjusted CSF age acceleration from Levine and Zhang epigenetic clocks

**
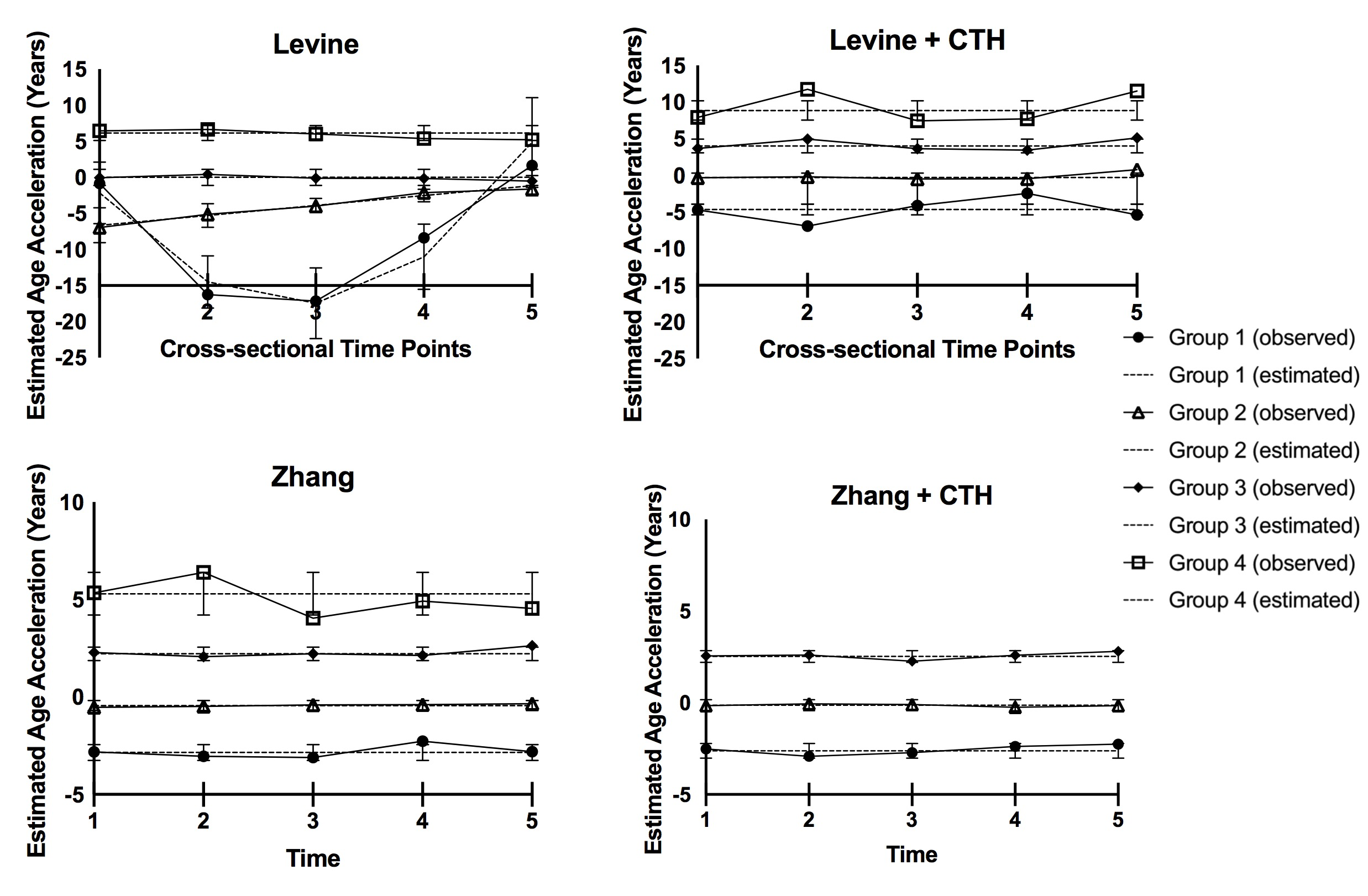
**

The above plots portray inferred trajectory groups and are not directly comparable as the group membership changes between plots; Sample size, n=273 at up to five time points (N=850 observations over 14 days post-aSAH condensed to 5 cross-sectional time points); Times listed correspond to cross-sectional time points (Time 1, Days 0 to 2; Time 2, Days 3 to 5; Time 3, Days 6 to 8; Time 4, Days 9 to 11; Time 5, Days 12 to 14 post-aSAH); CSF, cerebrospinal fluid; CTH, cell-type heterogeneity; aSAH, aneurysmal subarachnoid hemorrhage; Levine, Levine + CTH, and Zhang + CTH did not pass model QC evaluation as described above

**Table S2a.** Model selection for Horvath age acceleration group-based trajectory analysis

| **No. of groups** | **Model order** | **BIC1 (N=273)** | **BIC2 (N=850)** |
| --- | --- | --- | --- |
| 1 | 2 | -2258.71 | -2260.99 |
| 2 | 22 | -2179.14 | -2183.68 |
| 3 | 222 | -2162.26 | -2169.07 |
| 4 | 2222 | -2153.34 | -2162.42 |
| 5 | 22222 | -2157.16 | -2168.52 |
| 4 | 1111 | -2139.64 | -2145.89 |
| 4 | 0111 | -2139.64 | -2145.89 |
| 4 | 0011 | -2137.49 | -2143.17 |
| 4 | 0001 | -2134.77 | -2139.88 |
| 4 | **0000*** | **-2131.96** | **-2136.50** |

Table corresponds to Figure 6 (Horvath trajectory plot); **^*^**Best fitting model selected based on largest BICs; Model order, polynomial for each group (0=intercept, 1=linear, 2=quadratic); BIC, Bayesian Information Criterion; BIC1, computed based on participant sample size (N=273); BIC2, computed based on total number of observations (N=850)

**Table S2b.** Horvath age acceleration trajectory group posterior model quality control evaluation

| **Group** | **Estimated Group Membership (%)** | **Assigned Group Proportion (%)** | **Average Posterior Probability** | **Odds of Correct Classification** |
| --- | --- | --- | --- | --- |
| 1 | 3.1 | 2.9 | 0.91 | 321.6 |
| 2 | 41.4 | 42.1 | 0.86 | 8.5 |
| 3 | 40.4 | 41.0 | 0.80 | 5.7 |
| 4 | 15.1 | 13.9 | 0.85 | 30.8 |

Table corresponds to model 0000 identified above and plotted in Figure 6 (Horvath trajectory plot)

**Table S3a.** Model selection for CTH-adjusted Horvath age acceleration group-based trajectory analysis

| **No. of groups** | **Model order** | **BIC1 (N=273)** | **BIC2 (N=850)** |
| --- | --- | --- | --- |
| 1 | 2 | -2082.74 | -2085.07 |
| 2 | 22 | -1995.17 | -1999.84 |
| 3 | 222 | -1956.33 | -1963.34 |
| 4 | 2222 | -1946.49 | -1955.84 |
| 5 | 22222 | -1949.36 | -1961.04 |
| 5 | 00000 | -1928.74 | -1933.41 |
| 5 | 11111 | -1932.43 | -1941.19 |
| **4** | **0000*** | **-1921.50** | **-1927.34** |

Table corresponds to Figure 6 (Horvath + CTH trajectory plot); **^*^**Best fitting model selected based on largest BICs; Model order, polynomial for each group (0=intercept, 1=linear, 2=quadratic); BIC, Bayesian Information Criterion; BIC1, computed based on participant sample size (N=273); BIC2, computed based on total number of observations (N=850); CTH, cell-type heterogeneity

**Table S3b.** Horvath CTH-adjusted age acceleration trajectory group posterior model quality control evaluation

| **Group** | **Estimated Group Membership (%)** | **Assigned Group Proportion (%)** | **Average Posterior Probability** | **Odds of Correct Classification** |
| --- | --- | --- | --- | --- |
| 1 | 9.6 | 8.4 | 0.88 | 67.3 |
| 2 | 46.6 | 49.5 | 0.83 | 5.7 |
| 3 | 36.2 | 34.8 | 0.84 | 9.5 |
| 4 | 7.6 | 7.3 | 0.84 | 66.6 |

Table corresponds to model 0000 identified above and plotted in Figure 6 (Horvath + CTH trajectory plot); CTH, cell-type heterogeneity

**Table S4a.** Model selection for Hannum age acceleration group-based trajectory analysis

| **No. of groups** | **Model order** | **BIC1 (N=273)** | **BIC2 (N=850)** |
| --- | --- | --- | --- |
| 1 | 2 | -2444.36 | -2446.63 |
| 2 | 22 | -2390.31 | -2394.85 |
| 3 | 222 | -2382.63 | -2389.44 |
| 4 | 2222 | -2381.53 | -2390.62 |
| 5 | 22222 | -2384.19 | -2395.55 |
| 5 | 11111 | -2376.48 | -2385.00 |
| 4 | 1111 | -2374.62 | -2380.87 |
| 4 | 0011 | -2374.90 | -2380.58 |
| 4 | 2111 | -2373.67 | -2381.05 |
| 4 | 2211 | -2376.28 | -2384.23 |
| 4 | 2200 | -2374.78 | -2381.59 |
| 4 | 2000 | -2373.36 | -2379.04 |
| **4** | **2100*** | **-2372.18** | **-2378.43** |

Table corresponds to Figure 6 (Hannum trajectory plot); **^*^**Best fitting model selected based on largest BICs; Model order, polynomial for each group (0=intercept, 1=linear, 2=quadratic); BIC, Bayesian Information Criterion; BIC1, computed based on participant sample size (N=273); BIC2, computed based on total number of observations (N=850)

**Table S4b.** Hannum age acceleration trajectory group posterior model quality control evaluation

| **Group** | **Estimated Group Membership (%)** | **Assigned Group Proportion (%)** | **Average Posterior Probability** | **Odds of Correct Classification** |
| --- | --- | --- | --- | --- |
| 1 | 6.9 | 5.5 | 0.76 | 43.4 |
| 2 | 42.1 | 44.3 | 0.79 | 5.3 |
| 3 | 48.3 | 48.0 | 0.87 | 6.9 |
| 4 | 2.3 | 2.2 | 0.94 | 524.0 |

Table corresponds to model 2100 identified above and plotted in Figure 6 (Hannum trajectory plot)

**Table S5a.** Model selection for CTH-adjusted Hannum age acceleration group-based trajectory analysis

| **No. of groups** | **Model order** | **BIC1 (N=273)** | **BIC2 (N=850)** |
| --- | --- | --- | --- |
| 1 | 2 | -2153.02 | -2155.36 |
| 2 | 22 | -2056.65 | -2061.32 |
| 3 | 222 | -2040.83 | -2047.84 |
| 4 | 2222 | -2025.46 | -2034.80 |
| 5 | 22222 | -2029.76 | -2041.44 |
| 5 | 11111 | -2027.41 | -2036.17 |
| 5 | 00000 | -2023.34 | -2029.18 |
| 4 | 2100 | -2015.94 | -2023.37 |
| 4 | 2000 | -2014.88 | -2022.79 |
| **4** | **0000*** | **-2014.52** | **-2020.36** |

Table corresponds to Figure 6 (Hannum + CTH trajectory plot); **^*^**Best fitting model selected based on largest BICs; Model order, polynomial for each group (0=intercept, 1=linear, 2=quadratic); BIC, Bayesian Information Criterion; BIC1, computed based on participant sample size (N=273); BIC2, computed based on total number of observations (N=850); CTH, cell-type heterogeneity

**Table S5b.** Hannum CTH-adjusted age acceleration trajectory group posterior model quality control evaluation

| **Group** | **Estimated Group Membership (%)** | **Assigned Group Proportion (%)** | **Average Posterior Probability** | **Odds of Correct Classification** |
| --- | --- | --- | --- | --- |
| 1 | 5.3 | 4.0 | 0.85 | 101.8 |
| 2 | 38.6 | 37.0 | 0.86 | 9.7 |
| 3 | 40.7 | 45.1 | 0.79 | 5.6 |
| 4 | 15.4 | 13.9 | 0.82 | 25.75 |

Table corresponds to model 0000 identified above and plotted in Figure 6 (Hannum + CTH trajectory plot); CTH, cell-type heterogeneity

**Table S6a.** Model selection for Levine age acceleration group-based trajectory analysis

| **No. of groups** | **Model order** | **BIC1 (N=273)** | **BIC2 (N=850)** |
| --- | --- | --- | --- |
| 1 | 2 | -2685.99 | -2688.26 |
| 2 | 22 | -2632.01 | -2636.56 |
| 3 | 222 | -2622.54 | -2629.35 |
| 4 | 2222 | -2613.95 | -2623.04 |
| 5 | 22222 | -2621.71 | -2633.06 |
| 4 | 1111 | -2613.88 | -2620.12 |
| 4 | 0000 | -2618.81 | -2623.36 |
| 4 | 0011 | -2623.15 | -2628.83 |
| 4 | 2000 | -2615.08 | -2620.75 |
| 4 | 2200**^b^** | -2604.94 | -2611.76 |
| **4** | **2100*^, a^** | **-2602.60** | **-2608.85** |

Table corresponds to Figure S10 (Levine trajectory plot); **^*^**Best fitting model selected based on largest BICs; ^a^Model failed posterior quality control evaluation as shown in the table below; ^b^’Next best’ fitting model that also failed posterior quality control evaluation; Model order, polynomial for each group (0=intercept, 1=linear, 2=quadratic); BIC, Bayesian Information Criterion; BIC1, computed based on participant sample size (N=273); BIC2, computed based on total number of observations (N=850)

**Table S6b.** Levine age acceleration trajectory group posterior model quality control evaluation

| **Group** | **Estimated Group Membership (%)** | **Assigned Group Proportion (%)** | **Average Posterior Probability** | **Odds of Correct Classification** |
| --- | --- | --- | --- | --- |
| 1 | 2.2 | 2.2 | 0.85 | 257.4 |
| 2 | 24.2 | 22.3 | 0.78 | 11.2 |
| 3 | 52.9 | 55.7 | 0.81 | 3.9^a^ |
| 4 | 20.6 | 19.8 | 0.85 | 21.6 |

Table corresponds to model 2100 identified above and plotted in Figure S10 (Levine trajectory plot); ^a^Model did not pass QC because odds of correct classification <5

**Table S7a.** Model selection for CTH-adjusted Levine age acceleration group-based trajectory analysis

| **No. of groups** | **Model**  **order** | **BIC1 (N=273)** | **BIC2 (N=850)** |
| --- | --- | --- | --- |
| 1 | 2 | -2083.55 | -2085.58 |
| 2 | 22 | -2012.32 | -2016.38 |
| 3 | 222 | -1986.20 | -1992.30 |
| 4 | 2222 | -1988.05 | -1996.17 |
| 4 | 1111 | -1980.40 | -1985.98 |
| 4 | 1100 | -1977.84 | -1982.92 |
| 4 | 2200 | -1977.02 | -1983.11 |
| 4 | 2000**^b^** | -1972.76 | -1977.84 |
| **4** | **0000*^, a^** | **-1972.32** | **-1976.38** |

Table corresponds to Figure S10 (Levine + CTH trajectory plot); **^*^**Best fitting model selected based on largest BICs; ^a^Model failed posterior quality control evaluation as shown in the table below; ^b^’Next best’ fitting model that also failed posterior quality control evaluation; Model order, polynomial for each group (0=intercept, 1=linear, 2=quadratic); BIC, Bayesian Information Criterion; BIC1, computed based on participant sample size (N=273); BIC2, computed based on total number of observations (N=850); CTH, cell-type heterogeneity

**Table S7b.** Levine CTH-adjusted age acceleration trajectory group posterior model quality control evaluation

| **Group** | **Estimated Group Membership (%)** | **Assigned Group Proportion (%)** | **Average Posterior Probability** | **Odds of Correct Classification** |
| --- | --- | --- | --- | --- |
| 1 | 21.8 | 18.3 | 0.84 | 18.8 |
| 2 | 53.0 | 60.1 | 0.79 | 3.3^a^ |
| 3 | 20.4 | 17.2 | 0.79 | 15.1 |
| 4 | 4.8 | 4.4 | 0.85 | 109.6 |

Table corresponds to model 0000 identified above and plotted in Figure S10 (Levine + CTH trajectory plot); CTH, cell-type heterogeneity; ^a^Model did not pass QC because odds of correct classification <5

**Table S8a.** Model selection for Zhang age acceleration group-based trajectory analysis

| **No. of groups** | **Model order** | **BIC1 (N=273)** | **BIC2 (N=850)** |
| --- | --- | --- | --- |
| 1 | 2 | -1976.98 | -1979.26 |
| 2 | 22 | -1870.74 | -1875.28 |
| 3 | 222 | -1843.26 | -1850.08 |
| 4 | 2222 | -1834.89 | -1843.98 |
| 5 | 22222 | -1833.35 | -1844.71 |
| 5 | 11111 | -1825.26 | -1833.77 |
| 4 | 1111 | -1825.26 | -1833.77 |
| 4 | 11 | -1865.38 | -1868.79 |
| 4 | 1000 | -1817.55 | -1822.66 |
| **4** | **0000*** | **-1817.79** | **-1822.33** |

Table corresponds to Figure 6 (Zhang trajectory plot); **^*^**Best fitting model selected based on largest BICs; Model order, polynomial for each group (0=intercept, 1=linear, 2=quadratic); BIC, Bayesian Information Criterion; BIC1, computed based on participant sample size (N=273); BIC2, computed based on total number of observations (N=850)

**Table S8b.** Zhang age acceleration trajectory group posterior model quality control evaluation

| **Group** | **Estimated Group Membership (%)** | **Assigned Group Proportion (%)** | **Average Posterior Probability** | **Odds of Correct Classification** |
| --- | --- | --- | --- | --- |
| 1 | 17.5 | 17.22 | 0.85 | 25.77 |
| 2 | 52.14 | 54.21 | 0.87 | 6.212 |
| 3 | 27.37 | 26.01 | 0.88 | 20.24 |
| 4 | 2.99 | 2.56 | 0.87 | 209 |

Table corresponds to model 0000 identified above and plotted in Figure 6 (Zhang trajectory plot)

**Table S9a.** Model selection for CTH-adjusted Zhang age acceleration group-based trajectory analysis

| **No. of groups** | **Model order** | **BIC1 (N=273)** | **BIC2 (N=850)** |
| --- | --- | --- | --- |
| 1 | 2 | -1243.10 | -1245.62 |
| 2 | 22 | -1166.95 | -1171.98 |
| 3 | 222 | -1143.30 | -1150.84 |
| 4 | 2222 | -1145.97 | -1156.03 |
| 4 | 0011 | -1133.71 | -1140.00 |
| 5 | 00000 | -1131.97 | -1138.25 |
| 4 | 0000 | -1132.18 | -1137.21 |
| 3 | 100^b^ | -1132.15 | -1136.55 |
| **3** | **000*^a^** | **-1129.93** | **-1133.71** |

Table corresponds to Figure S10 (Levine + CTH trajectory plot); **^*^**Best fitting model selected based on largest BICs; ^a^Model failed posterior quality control evaluation as shown in the table below; ^b^’Next best’ fitting model that also failed posterior quality control evaluation; Model order, polynomial for each group (0=intercept, 1=linear, 2=quadratic); BIC, Bayesian Information Criterion; BIC1, computed based on participant sample size (N=273); BIC2, computed based on total number of observations (N=850); CTH, cell-type heterogeneity

**Table S9b.** Zhang CTH-adjusted age acceleration trajectory group posterior model quality control evaluation

| **Group** | **Estimated Group Membership (%)** | **Assigned Group Proportion (%)** | **Average Posterior Probability** | **Odds of Correct Classification** |
| --- | --- | --- | --- | --- |
| 1 | 24.77 | 13.55 | 0.89 | 23.98 |
| 2 | 47.8 | 71.06 | 0.64^a^ | 1.9^a^ |
| 3 | 27.43 | 15.38 | 0.92 | 32.52 |

Table corresponds to model 000 identified above and plotted in Figure S10 (Levine + CTH trajectory plot); CTH, cell-type heterogeneity; ^a^Model did not pass QC because odds of correct classification <5 and average posterior probability <0.7

**References**

1. Horvath S. DNA methylation age of human tissues and cell types. *Genome Biol*. 2013;14(10):R115. doi:10.1186/gb-2013-14-10-r115

2. Horvath S. Erratum to: DNA methylation age of human tissues and cell types. *Genome Biol*. 2015;16(1):96. doi:10.1186/s13059-015-0649-6

3. Hannum G, Guinney J, Zhao L, Zhang L, Hughes G, Sadda SV, Klotzle B, Bibikova M, Fan JB, Gao Y, Deconde R, Chen M, Rajapakse I, Friend S, Ideker T, Zhang K. Genome-wide Methylation Profiles Reveal Quantitative Views of Human Aging Rates. *Mol Cell*. 2013;49(2):359-367. doi:10.1016/j.molcel.2012.10.016

4. Levine ME, Lu AT, Quach A, Chen BH, Assimes TL, Bandinelli S, Hou L, Baccarelli AA, Stewart JD, Li Y, Whitsel EA, Wilson JG, Reiner1 AP, Aviv1 A, Lohman K, Liu Y, Ferrucci L, Horvath S. An epigenetic biomarker of aging for lifespan and healthspan. *Aging (Albany NY)*. 2018;10(4):573-591. doi:10.18632/aging.101414

5. Pidsley R, Y Wong CC, Volta M, Lunnon K, Mill J, Schalkwyk LC. A data-driven approach to preprocessing Illumina 450K methylation array data. *BMC Genomics*. 2013;14(1):293. doi:10.1186/1471-2164-14-293

6. Team RC. R: A language and environment for statistical computing. Published online 2018. https://www.r-project.org/

7. Zhang Q, Vallerga CL, Walker RM, Lin T, Henders AK, Montgomery GW, He J, Fan D, Fowdar J, Kennedy M, Pitcher T, Pearson J, Halliday G, Kwok JB, Hickie I, Lewis S, Anderson T, Silburn PA, Mellick GD, Harris SE, Redmond P, Murray AD, Porteous DJ, Haley CS, Evans KL, McIntosh AM, Yang J, Gratten J, Marioni RE, Wray NR, Deary IJ, McRae AF, Visscher PM. Improved precision of epigenetic clock estimates across tissues and its implication for biological ageing. *Genome Med*. 2019;11(1):54. doi:10.1186/s13073-019-0667-1

8. Zhang Q. DNA methylation based chronological age predictor. Published online 2019. doi:10.5281/zenodo.3369456
